# Developmental studies of the sublingual and mandibular salivary glands in Japanese quails (*Coturinx coturinx japonica*)

**DOI:** 10.1186/s12917-024-04355-7

**Published:** 2024-11-12

**Authors:** Mahmoud Osman Khalifa, Mahmoud Abd-Elkareem, Wafaa Gaber, Abdelmohaimen Mostafa Saleh

**Affiliations:** 1 Department of Anatomy and Embryology, Faculty of Veterinary Medicine, Aswan University, Aswan, 81528 Egypt; 2 Department of Cell and Tissues, Faculty of Veterinary Medicine, Assiut University, Assiut, Egypt; 3 Department of Anatomy and Embryology, Faculty of Veterinary Medicine, Assiut University, Assiut, Egypt

**Keywords:** Quail, Mandibular gland, Mucous, Sublingual salivary glands, Taste bud, Tubuloalveolar

## Abstract

**Background:**

The Japanese quail (*Coturinx coturnix japonica*) has a crucial role in the lives of humanity since the 12^th^ century and continues to play main roles in our industry and scientific research. The advantages that the Japanese quail has, such as heavy egg production and high-quality meat with low cholesterol and fat contents, Moreover, the Japanese quail is easily managed, with high feeding conversion, low cost of investment, and high rate of returns. Salivary glands are a part of the lingual apparatus that secretes serios and mucous saliva. Whereas, the saliva secretions have different roles in the food variation, apprehension, and moisture of food bolus. The morphological and cytochemical analysis are done on 20 healthy *Japanese* quail embryos of 6^th^, 10^th^, 11^th^, and 13^th^ days of incubation and 25 healthy quail chicks at hatching day old, 7^th^, 14^th^, 30^th^, and 60^th^ days old. These samples are investigated histologically, histochemically, and scanned by electron microscopy serially. Our purpose of the study is to highlight the area of the oropharyngeal salivary glands and their role in food variation, as few studies spoke about that in Japanese quail.

**Results:**

The primordia of the sublingual and mandibular salivary glands were noticed at the 6th and 10th days of the prehatching respectively as an epithelial bud. After hatching, both primordia were elongated and differentiated into secretory units. These glands were mucous polystomatic tubulo-alveolar paired glands, which were situated in the submucosa of the oropharyngeal floor (sublingual floor and paralingual grooves). The sublingual glands consisted of 3–5 lobes extended from the two Os ceratobranchial by their wide ends caudally, to beyond the median sulcus of the prefrenular part of the sublingual space rostrally. The taste buds were variable in size and position. The mandibular glands lay on the paralingual groove, which arose at the 10-day old embryo. The mandibular glands were located dorsomedial to the sublingual glands and extended longitudinally from the rostral border of the frenulum linguae to the caudal tips of the sublingual glands. The taste buds decreased in volume and number with advancing age.

**Conclusion:**

Overall, salivary glands increase in their alcianophilic activity of the secretions with advancing age, which indicates low PH within the secretory end pieces.

**Supplementary Information:**

The online version contains supplementary material available at 10.1186/s12917-024-04355-7.

## Highlights

• The oropharyngeal floor contains both the sublingual and mandibular salivary glands.

• The sublingual and mandibular salivary glands were compound tubuloalveolar paired glands.

• The sublingual salivary glands consist of non-branched 3–5 elongated lobes with common excretory duct.

• The primordia of the sublingual salivary gland developed at 6 days old embryo as an epithelial bud.

• The primordia of the mandibular salivary gland developed at 10 days old embryo.

• Both glands’ mucous secretion developmentally became with strong alcinophilic (acidic) contents.

• The surface taste buds decreased in volume and number with age advancing.

• Taste buds differed in size and position with taste pores from 8.2 to 12 μm.

**Supplementary Information:**

The online version contains supplementary material available at 10.1186/s12917-024-04355-7.

## Introduction

 Salivary glands are an auxiliary part of the lingual apparatus in birds, with a variety of cytochemical characteristics relating to the species’ food patterns. Several studies are examined the function, morphology, and histology of these structures [1–4]. However, in comparison to mammals, the glands play a minor function in saliva secretion. Saliva has two functions: antibacterial and moisture for food bolus [5]. Interestingly, many birds had glands that secrete mucous saliva [4, 6], but few birds have serous or seromucous glands [7]. Woodpecker mouth secretes sticky saliva, which aids in the adhesion of ants and insects [8].

Paired rostral sublingual glands and caudal paired mandibular salivary glands are reported in chickens [9] and Muscovy ducks [10]. These studies point out to the histology and histochemistry of the avian salivary glands [11, 12]. Of note, many taste buds are located on the soft and glandular epithelia of the oropharynx [13]. However, the taste bud system in the birds is less developer than the mammals and has two types of the taste buds [14]. But the histogenesis and histochemistry of either the oropharyngeal glandular system or the taste bud system during the pre- and post-hatching periods in male and female Japanese quail are not reported any more. So, the present study aims to investigate the developmental changes of the sublingual and mandibular salivary glands of Japanese quail.

## Materials and methods

The quails were obtained from a poultry farm of the Faculty of Agriculture, Assiut University, Assiut, Egypt. Forty-five healthy Japanese quails (Coturnix coturnix japonica) were divided into twenty prehatching embryos starting from the 6th day pre-hatching till hatching day (zero day old), then twenty-five chick quails at the 7, 14, 30, and 60th day post-hatching old. The eggs were collected within 1 week of laying and preserved in a refrigerator at 4 °C for ensuring the symmetrical aging of the specimens before placing into the incubator. Fertilized eggs were put on a forced-draft incubator (37.5° ±0.3 °C**/**60% RH). The oropharyngeal floor was incised and exposed, accordingly to [4].

### Gross double staining visualization

Three specimens from each age the seventh- and fourteenth-day-old quail chicks were double stained with either alcian blue and alizarin red or both following [4]. Briefly, the lower beak was fixed over night at room temperature in 95% ethanol after removing the skin and soft tissue. Then, the maceration process was done in many steps, as follows: Firstly, the adipose tissue was dissolved in 100% acetone for 48 h until the samples became clear. Next, we immersed the samples in 1% KOH for duration of 24 to 48 h. Consequently, the samples were incubated in 6% alcian blue/absolute alcohol for 48 to 72 h. After that, the samples were washed into two exchanges of 70% alcohol overnight. Thereafter, the clearing process was proceeded by 1% KOH overnight. Finally, the KOH1% was replaced with 1% KOH/alizarin red solution for 48 to 72 h. At that point, the clearing process with 1% KOH and the preservation process in 100% glycerol were done. Using the stereomicroscope (LEICAS6D), the measurements were done by ImageJ software (https://fiji.sc/).

## Histological and histochemical examinations

For paraffin sections and staining, three specimens of each age were used for the fixation, histological process, and decalcification, which were done [4]. After proper fixation, the samples were kept in 10% formic acid/formol saline overnight for the process of decalcification. Briefly, the samples were immersed into 1/10 volume formic acid/formol saline after clear washing in PBS to remove the excess fixative. When the sample became soft in texture, that ensured adequate decalcification of the bony and cartilaginous contents of the specimens. Notably, no more exposure for the decalcified solution that was harsh to the samples. After decalcification, the specimens were washed by distilled water, and then they dehydrated in ascending degrees of ethanol (70–100%). Then, samples were cleared in methyl benzoate to be immersed in paraffin wax stages I, II, and III. Lastly, the specimens were embedded in paraplast blocks (Sigma Aldrich) to have serial 5–6 μm cross, longitudinal, and frontal sections from the oropharyngeal floor that were cut by a LEICA 2155rm automatic microtome. The sections were stained with Harris hematoxylin and eosin stain [15]. Crossmon’s triple technique [16], Periodic acid-Schiff (PAS) [17], Alcian blue [18], and Alcian blue-PAS stain [17].

### Scanning electron microscope (SEM)

For scanning electron microscope (SEM) investigations of 10 and 13-day prehatching and 60-day post-hatching quail were used. The components of the floor of the oropharynx were washed several times in 0.1 M phosphate buffer at pH (7.2 ± 0.1). Post-hatching samples were rinsed with acetic acid at 2% and then fixed in a 4% glutaraldehyde solution for 24 h. Post-fixation was made in a 1% sodium tetroxide solution for two hours at 4 °C. The fixed samples were washed in 0.1 M phosphate buffer again at pH = (7.2 ± 0.1), then dehydrated in ascending grades of ethanol, followed by critical point-drying in liquid carbon dioxide. Specimens were mounted on aluminum stubs covered with carbon tabs and sputtered with gold. The prepared specimens were examined and photographed using JEOL scanning electron microscopy (JSM-5400) at an accelerating voltage of 15 kV in the electron microscope unit of Assiut University [19].

### Statistical analysis

The micrometrical measurements were taken by ImageJ software (https://fiji.sc/) and statistically analyzed by SPSS software.

## Results

### The sublingual salivary glands

**Fig. 1:**
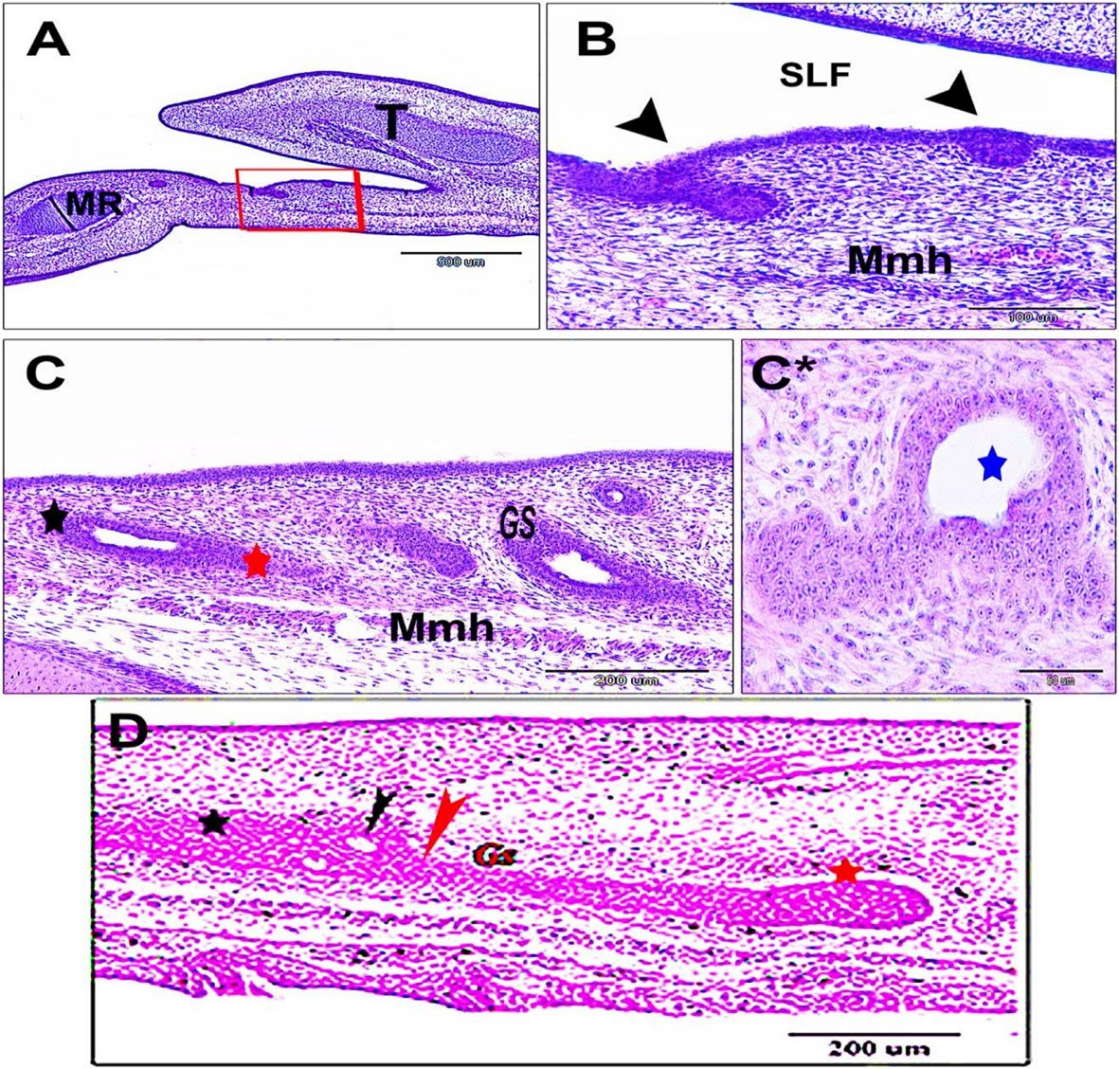
Photomicrographs of the sublingual floor (**A** & **B**). Sagittal sections of a 6-day old quail embryo, showing the primordia of the sublingual salivary glands (Rectangular shape, Fig. 1A). Note: the epithelial thickening of the sublingual floor mucosa (epithelial placode) (black arrowheads, Fig. 1B). (SLF) sublingual floor, (T) tongue, (MR) mandibular ramus, muscle mylohyoideus (Mmh) (**C**). A 10-day old quail embryo showing the glands sublingualis (Gs) have a canalized cord-like shape with two ends; rostromedial wide part (black star), and the caudolateral narrow part (red star). Notice: the muscle mylohyoideus (Mmh). (C*): Cross section showing canalization (blue star) within the gland and stratified lining epithelium of the same age. **D** The 11-day old quail embryo showing the extension of the cord of the gland sublingualis (Gs) with its rostromedial wide part (black star) covered by a concentric layer of mesenchyme and caudolateral narrow part (red star) uncovered by mesenchyme, and central constricted part (red arrowhead) small spaces have been shown (black arrowhead). H & E, Scale bars; A: 500 μm, B: 100 μm, C: 200 μm, C * : 50 μm, D: 200 μm

 The sublingual salivary gland primordia was seen in the 6-day-old quail embryo as an epithelial thickening of compact cellular mass (epithelial placode and prebud) within the soft sublingual floor epithelium of the oral floor (Fig. 1A and B). The highly proliferated epithelial masses extended from the bud to beyond the elongated cords at the 10-day-old quail embryo. These cords had two ends; a rostromedial wide end and a caudolateral narrow end. Canalization was observed in some cords (Fig. 1C and C*). With advancing age, the lobar cords of the sublingual salivary glands increased in their length and directed more caudally without branching at the 11-day-old quail embryo. The rostral end of the cord was broad, less proliferative, and encased by a concentric layer of mesenchyme, whereas the narrow caudolateral proliferative end was still uncovered, suggesting further growth and expansion. The cords were constricted nearly mid-distance. Due to cell growth competition and apoptosis, many vacuoles were obviously present within the rostral part of the cords, indicating the beginning of canalization (Fig. 1D). The canalization started rostrally (proximally) and progressed distally (caudally), and the glands extended without branching.

**Fig. 2:**
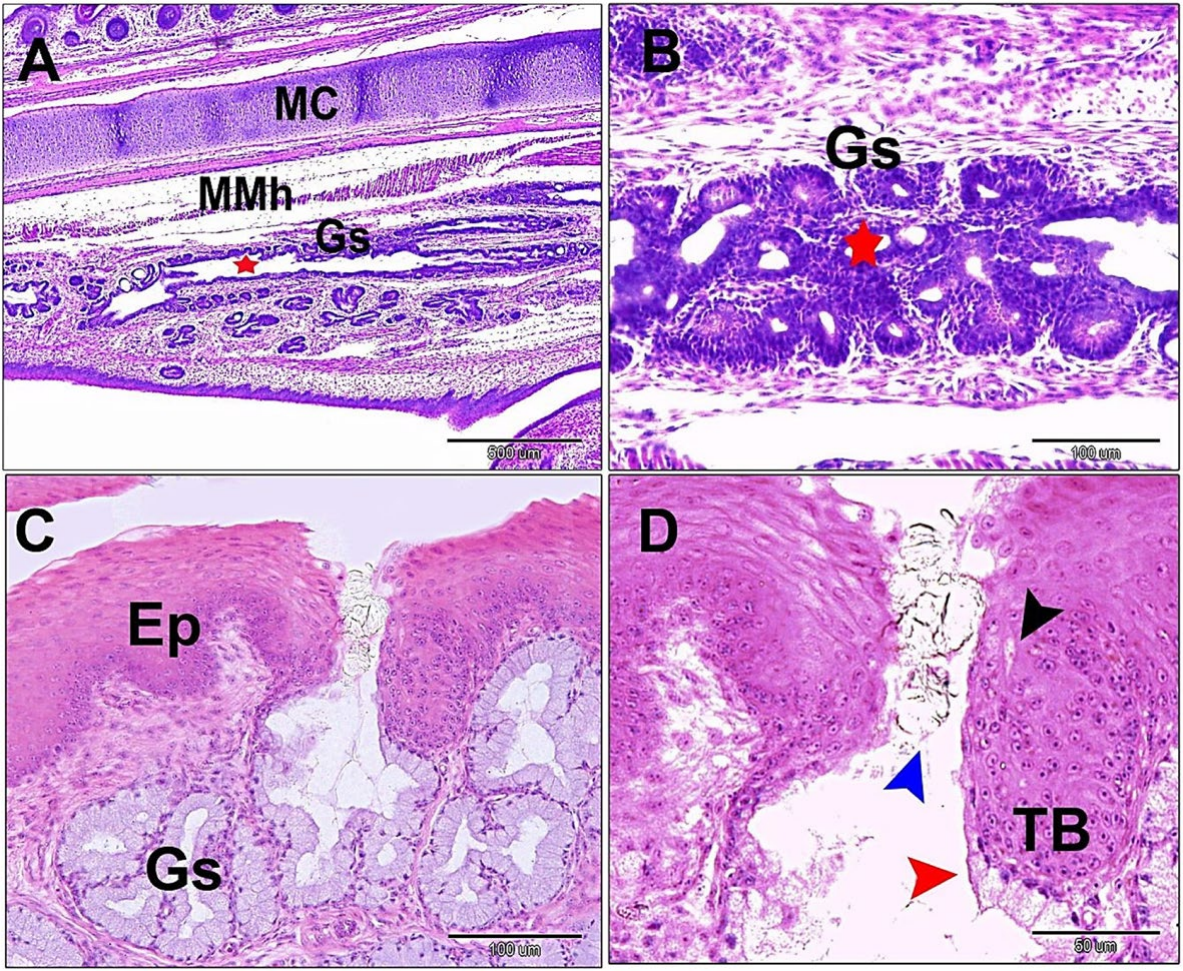
Photomicrographs of frontal sections in the sublingual floor of 13-day old quail embryo (**A**) straight common canal (red star), gland sublingualis (Gs). myelohyoid muscle (Mmh) and Meckelian cartilage (MC). **B** cellular exfoliation (red star) of the gland sublingualis. **C** & **D** Cross sections of a hatching quail chick; **C** sublingualis (Gs) surface epithelium (Ep). **D** simple columnar glandular epithelium (red arrowhead), infoldings in secretory end pieces (blue arrowhead). taste bud (TB), taste pore (black arrowhead). H & E, Scale bars; A: 500 μm, B: 100 μm, C: 100 μm, D: 50 μm

By the 13^th^ day of incubation, many vacuoles within the glandular end pieces coalesced together to form a single common canal rostrally. The lining of the glandular epithelium showed a transformation from stratified epithelium to simple glandular epithelium. The lumen was filled with a secretory-like substance, that represented the sloughed lining epithelium (Fig. 2A and B). The exfoliated substance was negative for PAS/Alcian blue. At the maturation stage, the secretory terminals presented more lining corrugation and cellular compaction. The lining epithelium showed low columnar cells with foamy cytoplasm and basal vesicular nuclei. Near the opening, the lining epithelium transformed from high columnar to low columnar and ends by stratified squamous epithelium (Fig. 2C and D).

**Fig. 3:**
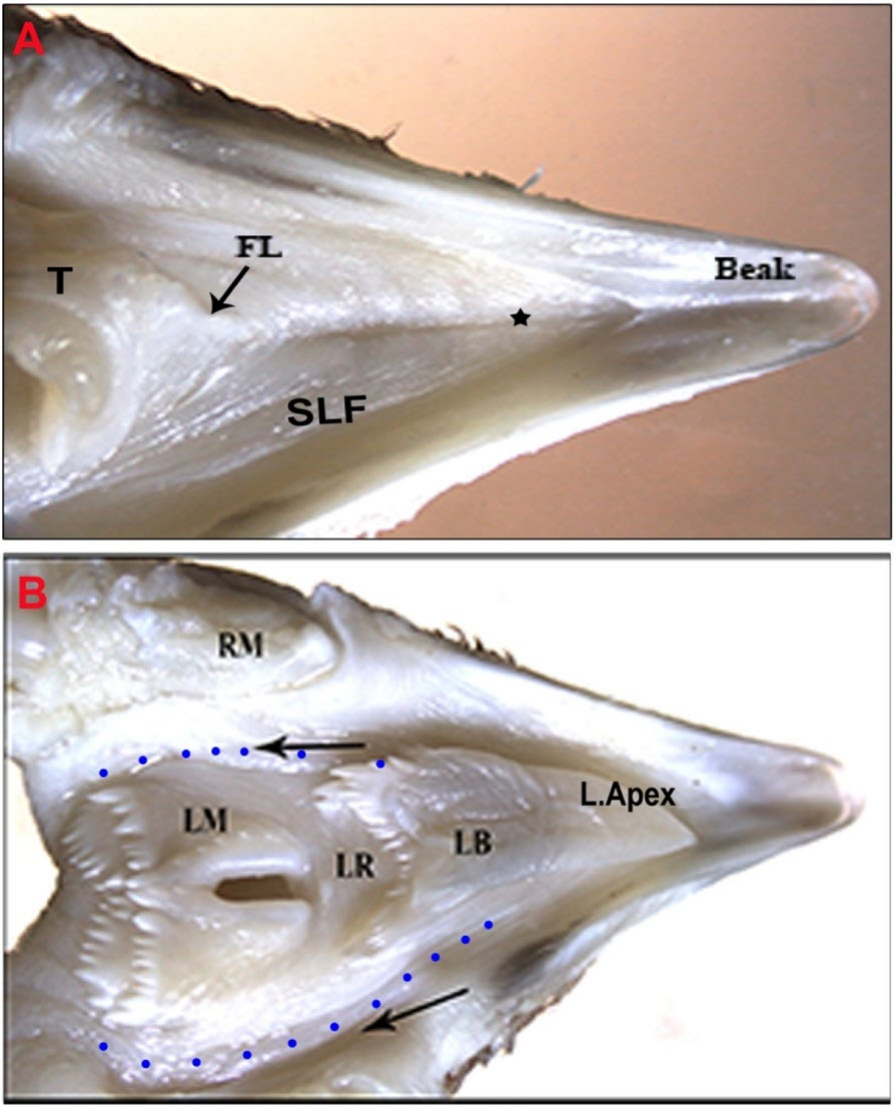
Photographs of a dorsal view of the oropharyngeal floor of a 14-day old quail chick. **A** sublingual floor (SLF) tongue (T), and prefrenular median sulcus (black star). frenulum linguae (FL). (X 6.3). **B** Photograph showing paralingual grooves (black arrows) and mandibular salivary glands (blue dots). Note, Ramus mandibularis (RM), lingual apex (L.Apex), lingual body (LB), lingual root (LR), and laryngeal mound (LM). (X 6.3)

**Fig. 4:**
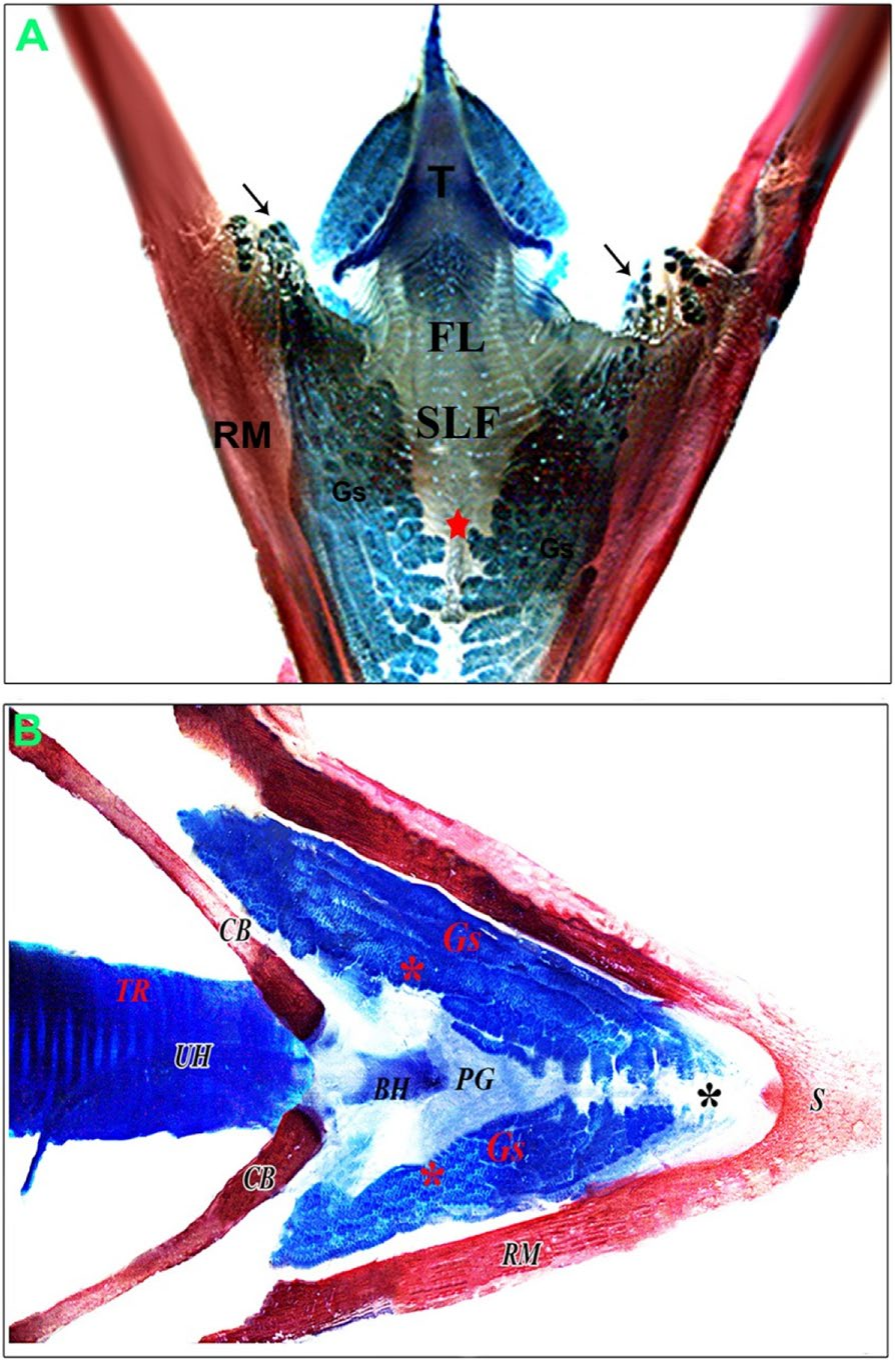
After maceration of 14-day-old quail chick (**A**) Dorsal view of the oral floor after tongue (T) reflection showing sublingual floor (SLF) and prefrenular median sulcus where sublingual salivary gland open (red star). Note, frenulum linguae (FL). (Alzarin red & alcian blue stains, X 6.3). **B** ventral view of the lower beak of a 14-day-old quail chick showing gland sublingualis (Gs) extends from symphysis mandibularis (S) beyond the first parts of the ceratobranchialia (CB) and ramus mandibularis (RM). Note the basihyoid (BH), paraglossal (PG), trachea (TR), urohyale (UH), and central constricted part (red asterisks). Sublingual groove (black asterisk). (Alizarin red & alcian blue X 6.3)

 Gross anatomical examination of 14-day-old chicks revealed that the sublingual glands were paired, consisting of 5–7 parallel elongated lobes with broad blind ends that directed rostromedially and narrow ends directed caudolaterally (Fig. 4). The glandular lobes extended longitudinally from the mandibular symphysis rostrally within the sublingual floor to beyond the level of the proximal part of the ceratobranchial. The lobes were constricted at the level of the basihyal (Fig. 3), where they supported the laryngeal mound; therefore, it was suggested the significant size of the larynx. The lobes decreased in the lobation number at that portion (Fig. 4). The lobes were numerous rostrally and few (Fig. 3) and (Fig. 4). There was an asymmetrical bilateral glandular lobe number along the same side.

**Fig. 5:**
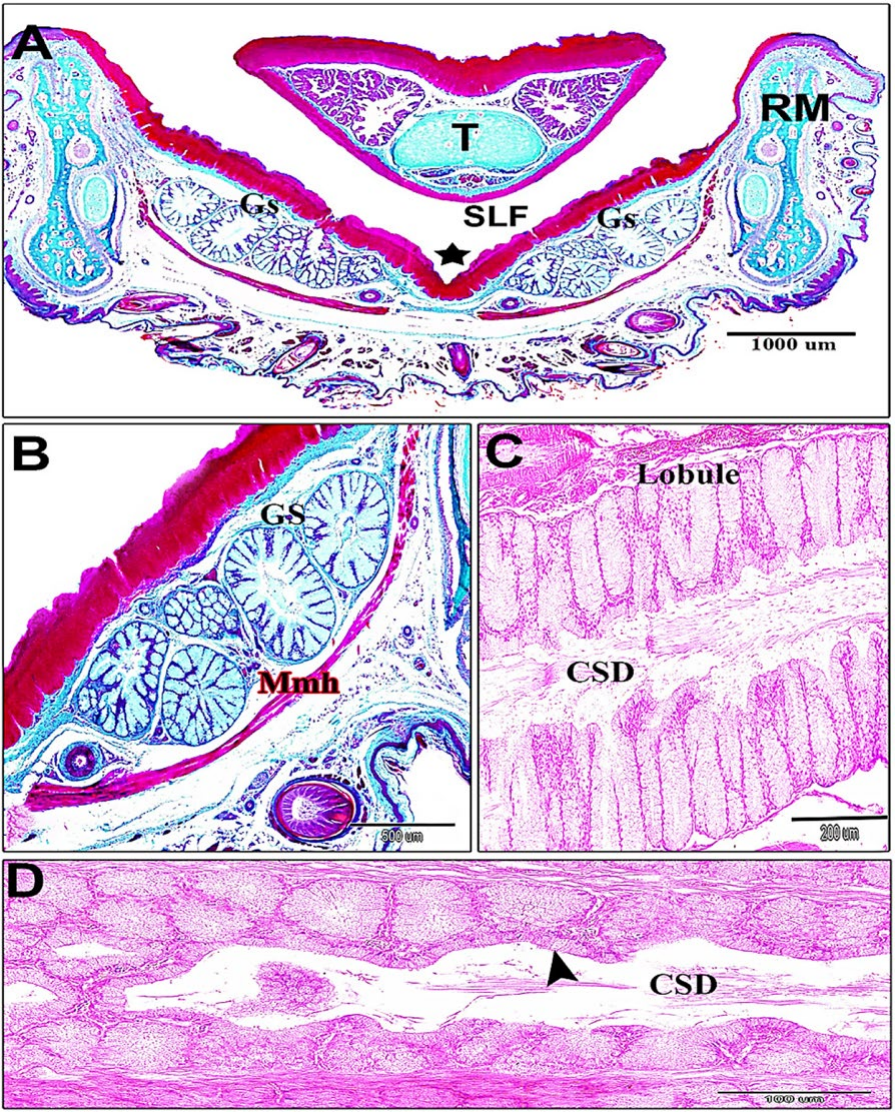
A 30-day-old quail (**A** & **B**). Paired gland sublingualis (Gs), which was composed of variable 5–7 lobules in each side at the oral part; lying in the submucosa and supported ventrolaterally by muscle mylohyoideus (Mmh). Note: median sulcus (black star), ramus mandibularis (RM), sublingual floor (SLF), and tongue (T). (Crossmon’s trichrome stain, scale bars; A: 1000 μm, B: 500 μm). **C** & **D** Lobular structure of gland sublingualis composed of compound tubuloalveolar secretory units open into common secretory duct (CSD), which was lined by simple columnar epithelium (black arrowhead). (H & E stain, scale bars; C: 200 μm, D: 100 μm) Fig. 6. A 30-day-old quail. The left side showing gland sublingualis (Gs) consists of 3 lobes (A & B), while the right side shows 5 different-sized lobes: 2 larger ones, 2 moderate ones, and a smaller one in (**A** & **C**). Note: muscle mylohyoideus (Mmh), paralingual groove (Plg), gland mandibularis (Gm), ramus mandibularis (RM), laryngeal mound (LM). (Crossmon’s trichrome stain, scale bars: A: 1000 μm, B & C: 500 μm). (D & E): Autonomic ganglion (AG) adjusts to the glands sublingualis. (H & E stain, scale bars; D: 100 μm; E: 50 μm)

**Fig. 6:**
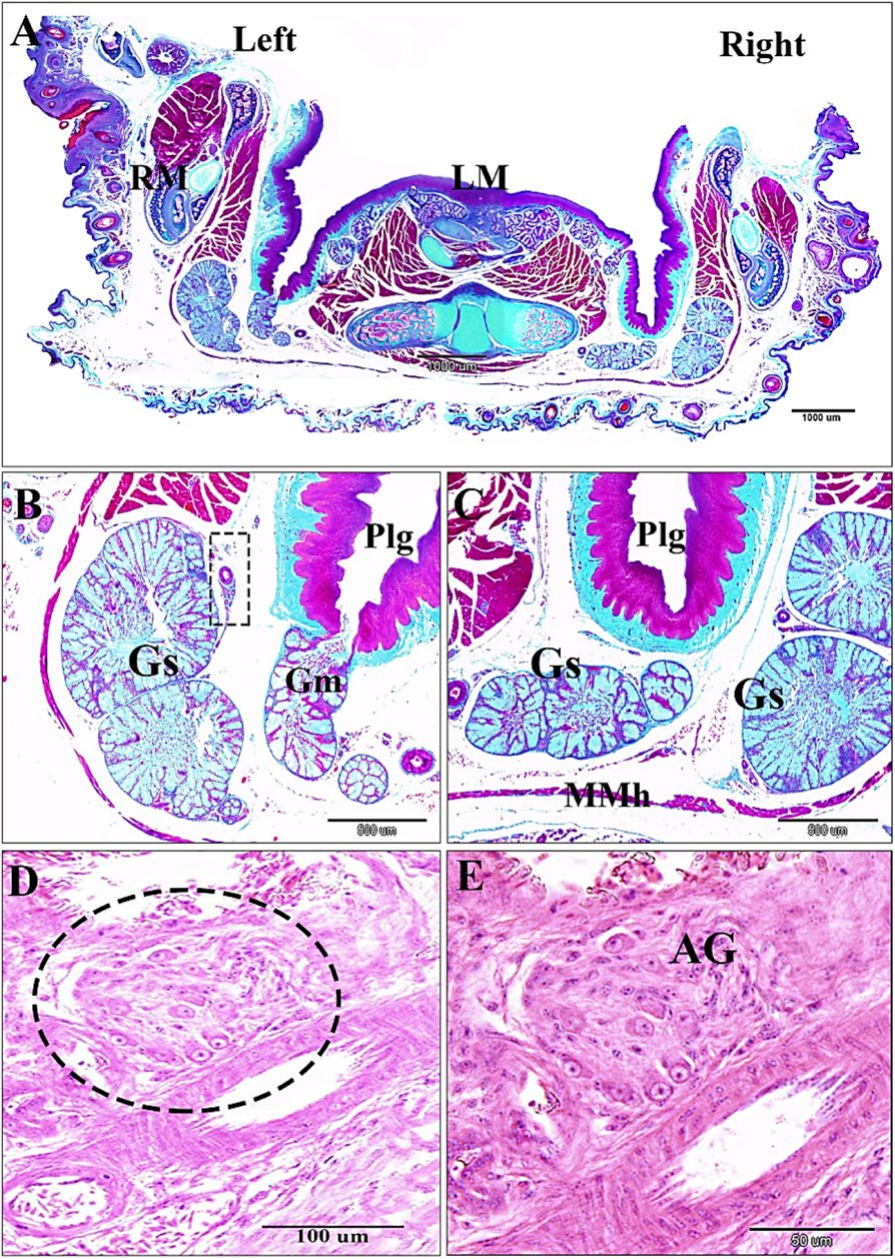
A 30-day-old quail. The left side showing gland sublingualis (Gs) consists of 3 lobes (**A** & **B**), while the right side shows 5 different-sized lobes: 2 larger ones, 2 moderate ones, and a smaller one in (**A** & **C**). Note: muscle mylohyoideus (Mmh), paralingual groove (Plg), gland mandibularis (Gm), ramus mandibularis (RM), laryngeal mound (LM). (Crossmon’s trichrome stain, scale bars: A: 1000 µm, B & C: 500 µm). **D** & **E** Autonomic ganglion (AG) adjusts to the glands sublingualis. (H & E stain, scale bars; D: 100 µm; E: 50 µm)

**Fig. 7:**
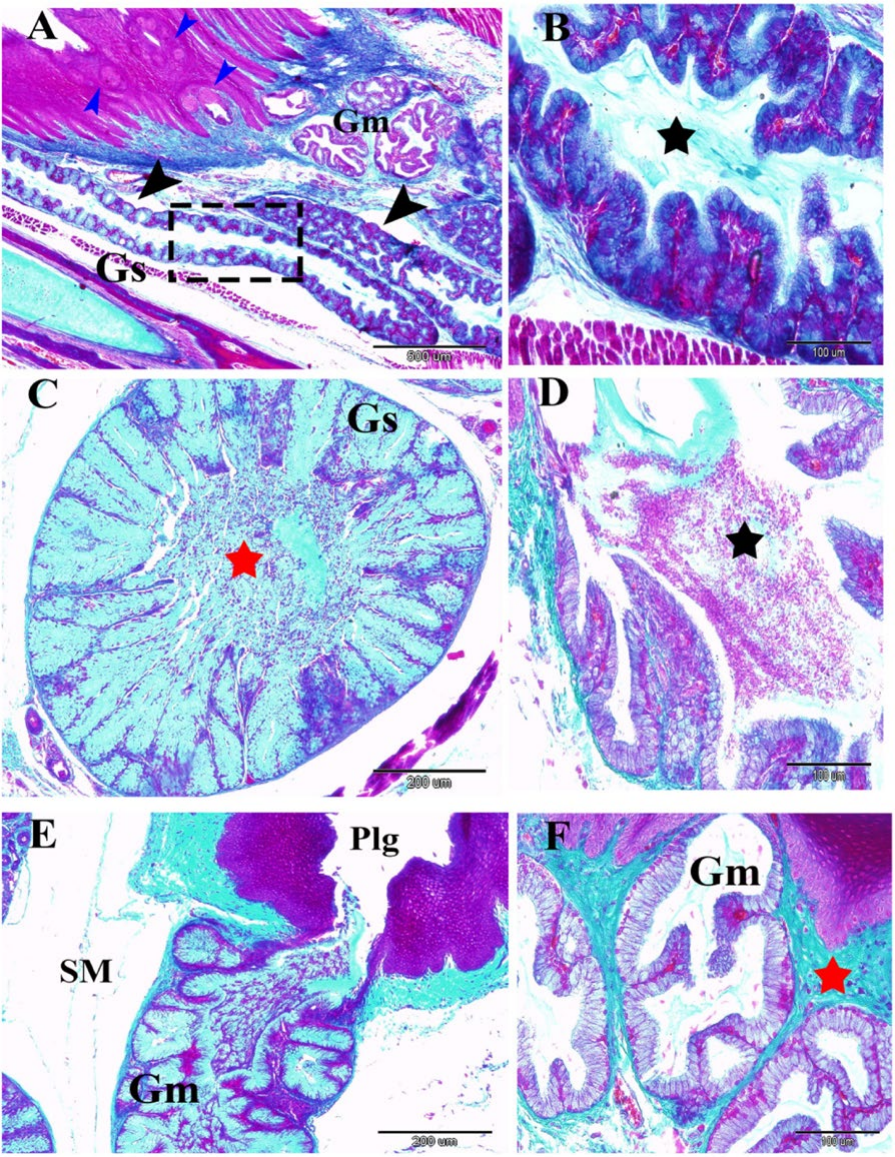
A 30-day-old quail chick. **A** Many taste buds within the epithelium (blue arrowheads), the gland sublingualis (black arrowheads), and the lobar structures of the gland sublingualis (black dash rectangular shape). **B** The lobar structure of the gland sublingualis with a central lumen (Black star) (**C**) Cross sections of the gland sublingualis (Gs) at the pharyngeal level with wide central lumen (red star) filled with secretion. **D** Apocrine secretion, the cellular content depris (red color), and secretion (greenish color). **E** Apocrine secretion of the gland mandibularis (Gm) poured into paralingual groove (Plg) with cellular contents and secretions. Note the submucosa (SM). (F): Secretory endpieces with thick connective tissue septa (red star) of the gland mandibularis (Gm). (Crossmon’s trichrome stains, scale bars; A: 500 µm, B, D & F: 100 µm, C & E: 200 µm)

The post-hatching histological studies at 14-and 30-day old quail chicks, suggested that the sublingual salivary glands were non-branched compound tubuloalveolar mucous glands that were supported ventrolaterally by muscle mylohyoideus, and a thin connective tissue stroma. The median prefrenular sulcus of the sublingual space was separated between two glandular masses rostrally. The secretory endpieces of the sublingual salivary glands of each lobule drained their secretion directly into a common secretory duct that opened into the soft sublingual floor separately or collectively with other canals (polystomatic). The ductal epithelial lining was simple, tall columnar cells. The ductal canal was narrow near the excretory surface epithelium (Fig. 5A-D). Histological data confirmed that the lobations at the level of the sublingual floor were 5–7 bilateral (Fig. 5A). But, caudally, the lobations were on the right side as two larger, two medium-sized, and one smaller lobes. The left side had three lobes; two larger and one smaller. The common canals of the sublingual glands were narrower rostrally than the caudal lobes (Figs. 6A-C and 7A-B). The autonomic ganglion that embedded among secretory lobules (Fig. 6D and E). The mucous of the sublingual salivary glands were shown the apocrine mode of secretion with cellular and nuclear contents that were detached within the lumen (Fig. 7C-D).

**Fig. 8:**
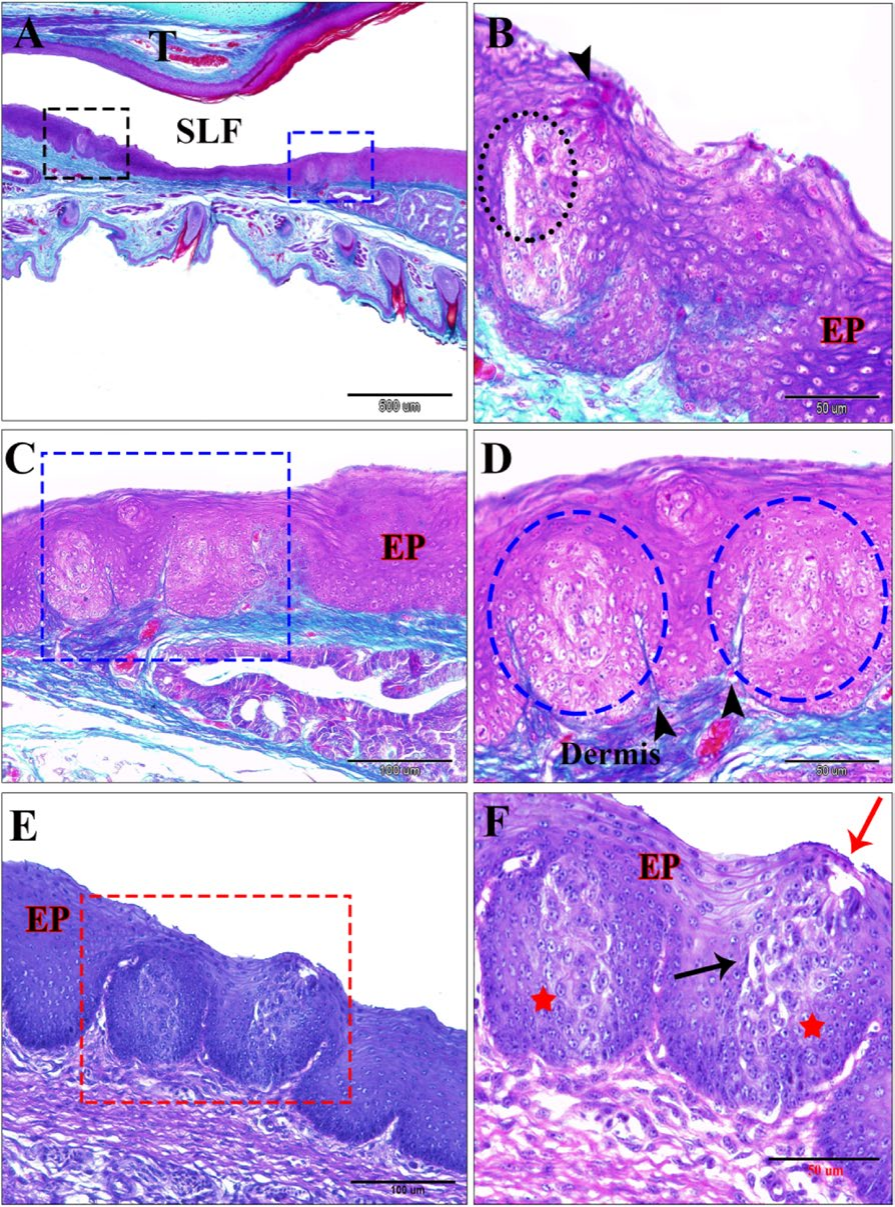
**A**-**D** A 14-day-old quail chick showing the types of barrel-shaped taste buds with taste pores. **A** Taste buds associated with salivary gland openings (black rectangular shaped) and surface epithelial taste buds (blue rectangular shaped). Note the tongue (T) and sublingual floor (SLF). **B** Showing taste pores (black dotted circle), dark stained cells (black arrowhead), and epithelium (Ep). **C** & **D** surface epithelial taste buds (blue dotted circle), with deep dermal papillae (black head arrows). (Crossmon’s trichrome stains, scale bars; A: 500 µm, B, D: 50 µm & C: 100 µm). **E** & **F** A 60-day-old quail showing the opening of the gland sublingualis (red arrow) arose from the epithelium of the sublingual floor, taste bud (red rectangle), taste pores (black arrow), the basal epithelial highly mitotic cells (red star), and the surface epithelium (Ep). (PAS & Hx stain, scale bars, E: 100 µm & F: 50 µm)

Two types of taste buds were found to have a barrel shape (Fig. 8A-B); firstly, the surface epithelial taste-buds were characterized by deep dermal papillae within the epithelium and deeply stained central cells assumed to be gustatory cells (Fig. 8C-D). Secondly, the taste buds-associated salivary gland opening was surrounded by highly mitotic polygonal basal cells within the barrel-shaped structure (Fig. 8E-F).

**Fig. 9:**
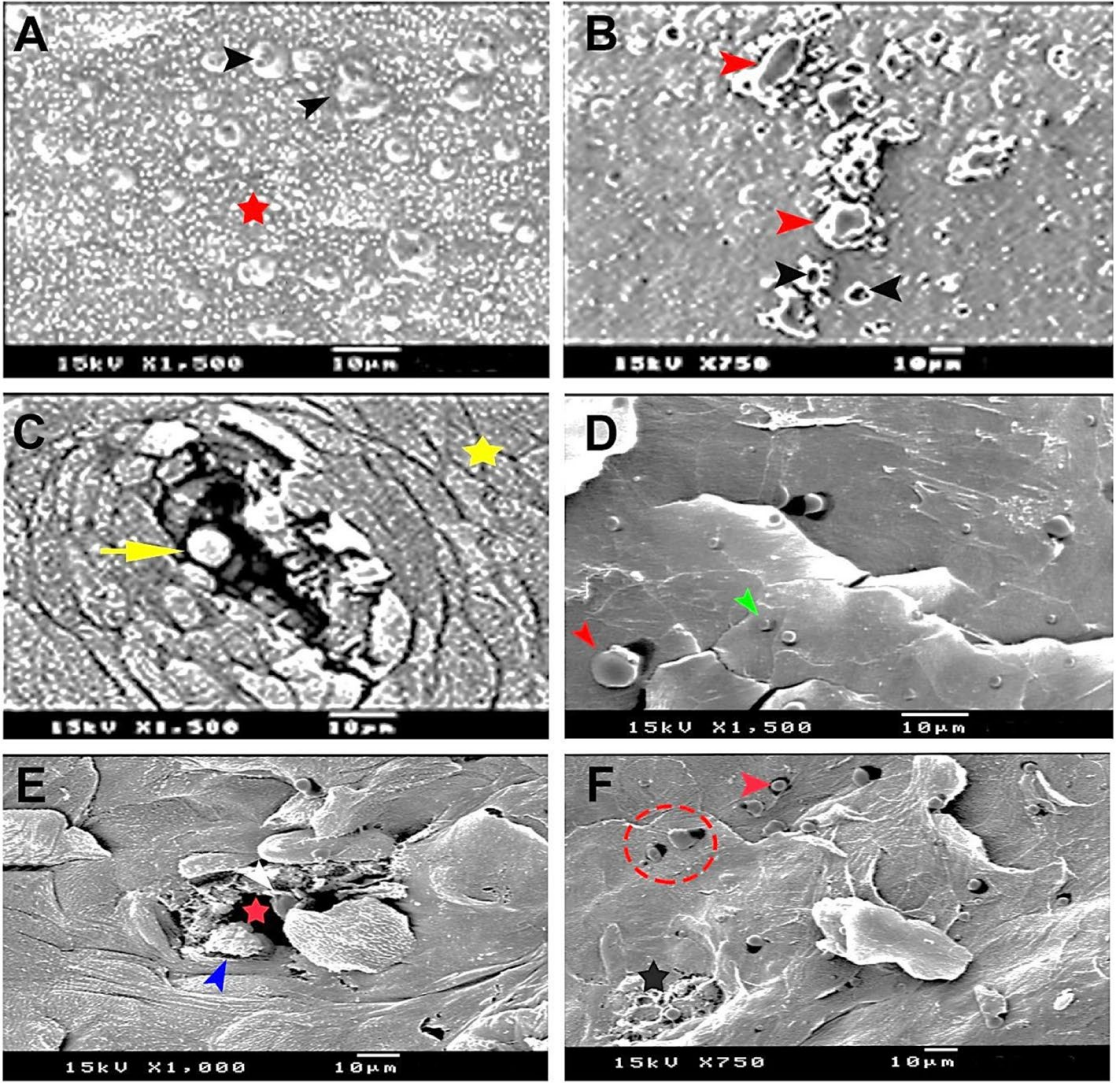
Scanning electron micrographs of sublingual floor surface (**A**) 8 days prehatching, showing dome-shaped mushroom-like taste buds (black arrowheads), sublingual floor (red star) (X 1500). **B** 10 days pre-hatching, showing sublingual salivary glands (black arrowheads) taste buds (red arrowheads). (X 750). **C** 13-day pre-hatching showing the opening of the sublingual salivary taste bud (yellow short arrow) and epithelium (yellow star) (X 1500). **D** 30-day-old chick showing taste buds (red arrowhead) surface taste buds (green arrowhead). **E** & **F** 60 days post-hatching quail; (**E**) sublingual salivary gland (red star), mucous debris adjacent to the opening's rim (blue arrowhead), large-sized taste buds associated with salivary gland (white arrowhead) (X 1000). **F** Showing the taste buds associated with the sublingual surface surrounded with pits (red dotted circle and red arrowhead), others associated with mucous (black stars). Note the decrease in the number of the small surface taste bud type

SEM (Fig. 9) and morphometrical measurements (Table 1) revealed that at 8 days old quail embryo, the soft sublingual floor was full of numerous domes, mushrooms, or spherical-like taste buds with various sizes, defined into three different sizes that were categorized into larger, moderate, and smaller taste buds, which ranged from (40, 12, and 6) um^2^, respectively. Also, the data showed some large-sized taste buds were nearly fused together (Fig. 9A). By the age of the 10th day old embryo, the taste buds had a mean surface area of 36 um^2^ and the sublingual salivary glands opening diameter was 5 μm. By the time progression, the taste buds decreased in number and size. However, some of them fused together to form a large one of 144 um^2^ (Fig. 9B). With age advancing (13 days pre-hatching), the sublingual gland opening increased in diameter and reached 28 μm with no secretion. The taste bud-associated salivary glands could be seen, with a surface area that was equal to 35 um^2^ (Fig. 9C). At 30- and 60-day old chicks, we found many various-sized taste buds that were measured as large size (32 um^2^), medium size (12 um^2^), and small size (9 um^2^), and their taste pores varied from 8.2 to 12 μm. These taste buds emerged through the scales of the surface of the sublingual floor epithelium, and mucous secretion exited via the salivary gland opening (Fig. 9D-F). The data indicated that the taste buds were different in size and distribution.

**Table 1:** Showing the average diameter (um) of the sublingual salivary gland openings (SGO), the taste buds openings (TBO) and taste buds surface area (TBA) (um^2^) on oropharyngeal floor of the Japanese quails

Item	SGO (um)	TBA (um^2^)	TBO (um)
Age			
8 days embryo	-	Small sized (6 ± 0.01) Medium sized (12 ± 0.05) Large sized (40 ± 0.015)	-
10 days embryo	5 ± 0.02	Decreased in number and some fused to be 144 ± 0.03	-
13 days embryo	28 ± 0.15	35 ± 0.012	-
Hatching chick	30 ± 0.1	-	11 ± 0.023
7 days chick	36 ± 0.04	-	12 ± 0.1
30- & 60-days chick	45 ± 0.08	Small sized (9 ± 0.06) Medium sized (12 ± 0.013) Large sized (32 ± 0.02)	8.2 ± 0.35

**Fig. 10:**
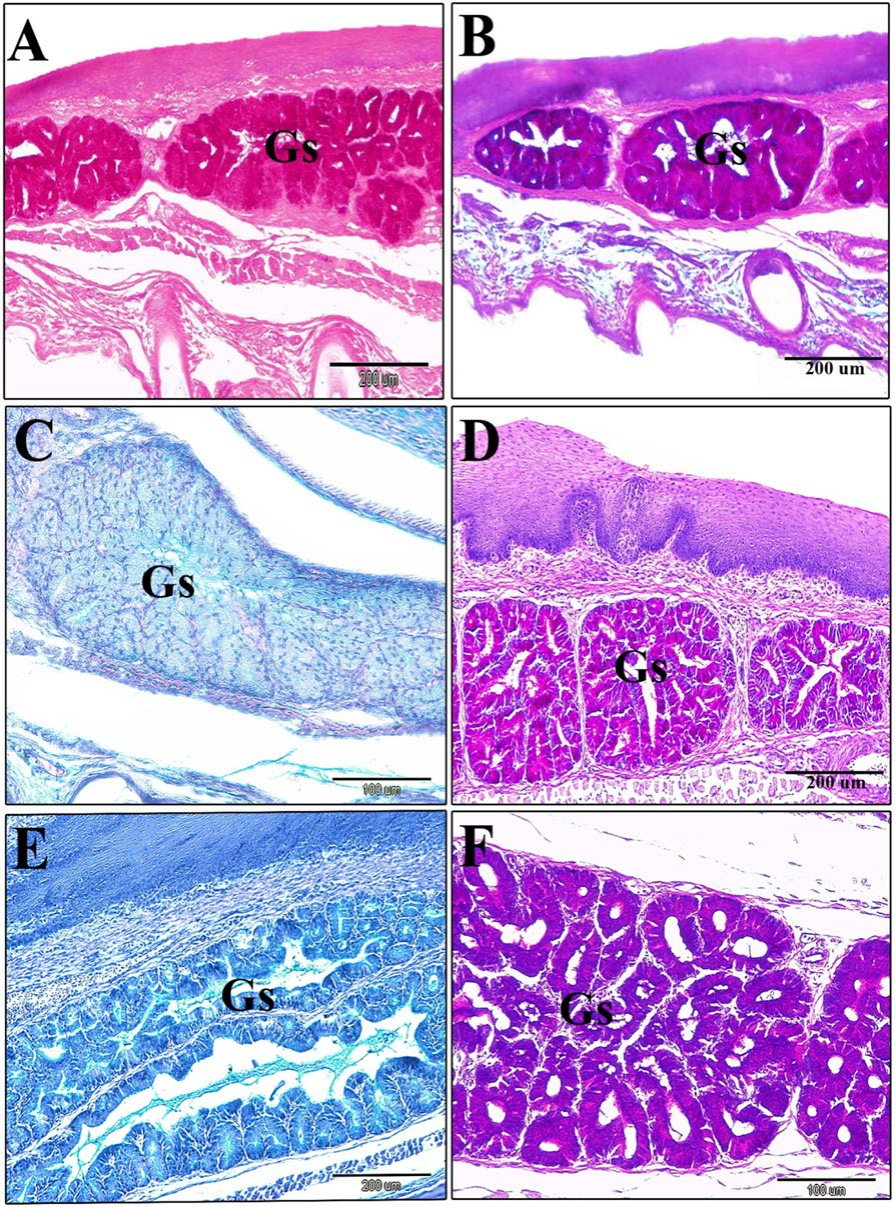
A newly hatching quail chick. **A** Showing the gland sublingualis is strong positive for PAS stain. (PAS stain, scale bar 200 µm). **B** Gland sublingualis is strong positive for combined AB/PAS stains. (AB/PAS stains, scale bar 200 µm). **C** Gland sublingualis weakly positive to AB stain, scale bar 100 µm). **D** 7-day-old quail chick showing the gland sublingualis strong positive to PAS stain. (PAS stain, scale bar 200 µm). **E** A 7-day-old quail chick showing the gland sublingualis moderately positive to AB stain. (AB stain, scale bar 200 µm). **F** A sagittal section of a 7-day-old quail chick showing the gland sublingualis is strongly positive for combined AB/PAS stains (purple coloration). (AB/PAS stains, scale bar 100 µm)

**Fig. 11:**
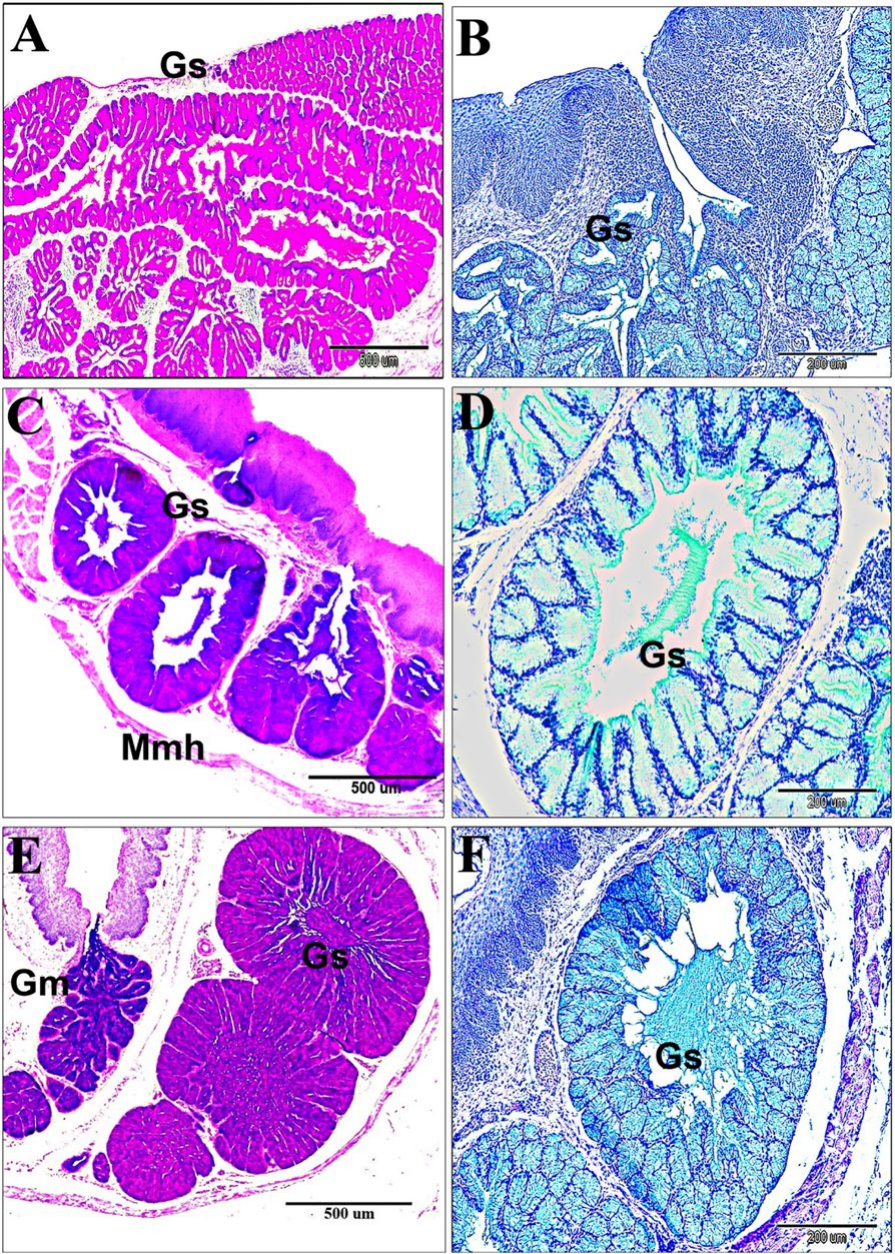
A 14-day (**A** & **B** & **C**); a 30-day (**D** & **E**); and a 60-day (**F**) old quail chick. **A** Gland sublingualis very strong positive to PAS stain. (PAS stain, scale bars 500 µm). **B** Gland sublingualis moderately positive to AB stain (AB stain, scale bars 200 µm). **C** very strong positive to combined AB/PAS stains. (AB/PAS stain, scale bars 500 µm). **D** & **E** Photomicrograph of a cross-section in the oropharyngeal floor showing the gland sublingualis strong positive to AB stain and very strong positive to combined AB/PAS stains (purple coloration), respectively. (D: AB, scale bars 100 µm; E: AB/PAS, scale bars 500 µm). **F** Cross section showing the gland sublingualis very strong alcinophilic to AB stain and the same result with combined AB/PAS stains. (AB stain, scale bar 200 µm)

Histochemical analysis of the sublingual glands (Table 2A) showed a strong positive reaction with both PAS and combined AB/PAS stains, while weak reaction to AB stain in the newly hatched quail chick (Fig. 10A-C). In the seventh-day-old chick, the sublingual glands showed a moderate positive reaction to alcian blue and a strong reaction to PAS and combined AB/PAS stains (Fig. 10D-F). However, in a 14-days-old quail chick, these glands showed a moderate positive reaction to AB, a very strong positive reaction to PAS, and combined AB/PAS stains (Fig. 11A-C). In 30-day-old quail, the sublingual glands showed a strong positive reaction to AB stain and a very strong positive reaction to combined AB/PAS stains (purple coloration), but contrary, negative results to PAS stain were shown (Fig. 11D-E). The 60-day-old quails and the glands were shown negative results to PAS stain and a very strong positive reaction to AB stain, and an alcianophilic reaction to AB/PAS stains (greenish blue coloration) (Fig. 11F).

**Table 2:** Showing the histochemical properties of the sublingual salivary glands (Gs) & mandibular salivary glands (Gm) in quail chicks

(A) Sublingual salivary gland						(B) Mandibular salivary gland
	Zero day	7 days	14 days	30 days	60 days	30 days
**AB**	**+/-**	**++**	**++**	**+++**	**++++**	**++++**
**PAS (Magenta)**	**+++**	**+++**	**++++**	**-**	**-**	**++++**
**AB/PAS** **Purple**	**+++**	**+++**	**++++**	**++++**	**++++** Alcinophilic (greenish-purple)	**++++** Alcinophilic (greenish-purple)

### The mandibular salivary glands

**Fig. 12:**
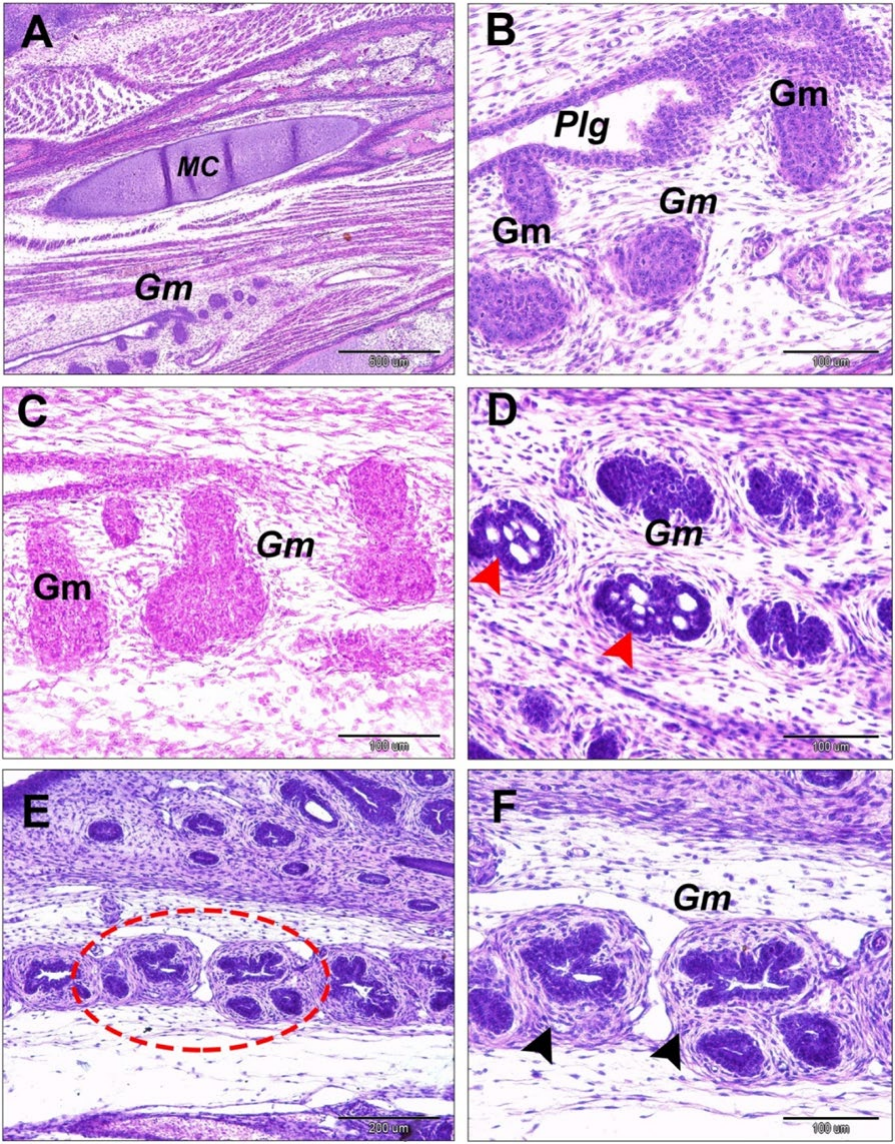
**A** & **B** Gland mandibularis (Gm) buds and cords of a 10-day-old quail embryo originated from epithelial of the paralingual groove (Plg). Note the meckelian cartilage (MC). **C** Expansion of the cord of an 11-day-old quail embryo. **D** Canalization of the expanded cord of a 12-day-old embryo and thin mesenchymal interaction (red arrowheads). **E** & **F** Thick concentric mesenchymal layers surrounded the branched cord of a 13-day-old embryo with more branched endpieces (black arrowheads). (H & E stains, scale bars; A: 500 µm, B, C, D & F: 100 µm, E: 200 µm)

**Fig. 13:**
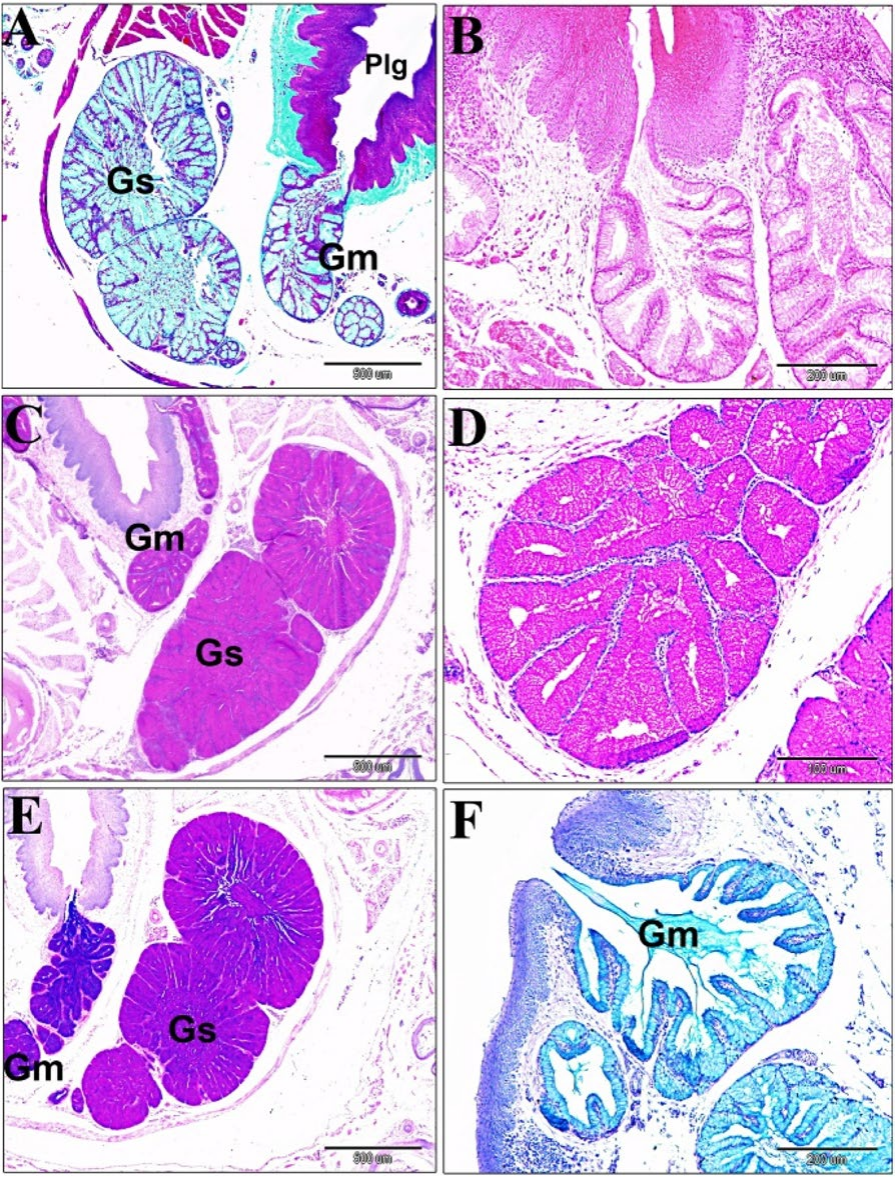
A 30-day-old quail (**A**) gland mandibularis (Gm) lies dorsomedially to gland sublingualis (Gs) and opens ventrally into paralingual groove (Plg). (Crossmon’s trichrome, scale bar 500 µm). **B** Showing the gland mandibularis (Gm) composed of compound tubule-alveolar secretory endopices lies in the submucosa. (H & E, scale bar 200 µm). **C** & **D** Gland mandibularis (Gm) and the gland sublingualis (Gs) very strong positive to PAS stain (PAS, scale bar; C: 500 µm, D: 100 µm). **E** Gland mandibularis (Gm) and the gland sublingualis (Gs) are very strong positive to combined AB/PAS stains. (AB/PAS stains, scale bar: 500 µm). **F** Gland mandibularis (Gm) very strong positive to AB stain. (AB stain, scale bar: 200 µm)

The mandibular glands appeared in a 10-day-old embryo that arose from the epithelium of the paralingual groove. Interestingly, the developmental characterization of the gland was like the sublingual glands in all stages. However, the mandibular glands were different in the branching process of the glandular endpieces (Fig. 12A-F). The glands extended caudally to the frenulum linguae, lying within the floor of the paralingual grooves (Fig. 3B). They were paired compound tubuloalveolar in the tunica submucosa of the paralingual grooves of the oropharynx. The mandibular glands extended longitudinally from the rostral border of the frenulum linguae to the caudal tips of sublingual glands. They lay on the dorsomedial border of the sublingual glands and opened into the ventral or medial aspect of the paralingual groove (Fig. 13A-B). The mode of the secretion resembled the sublingual gland. Moreover, the glandular acini were separated with thick connective tissue septa (interlobular connective tissue). (Fig. 7E-F).

**Fig. 14:**
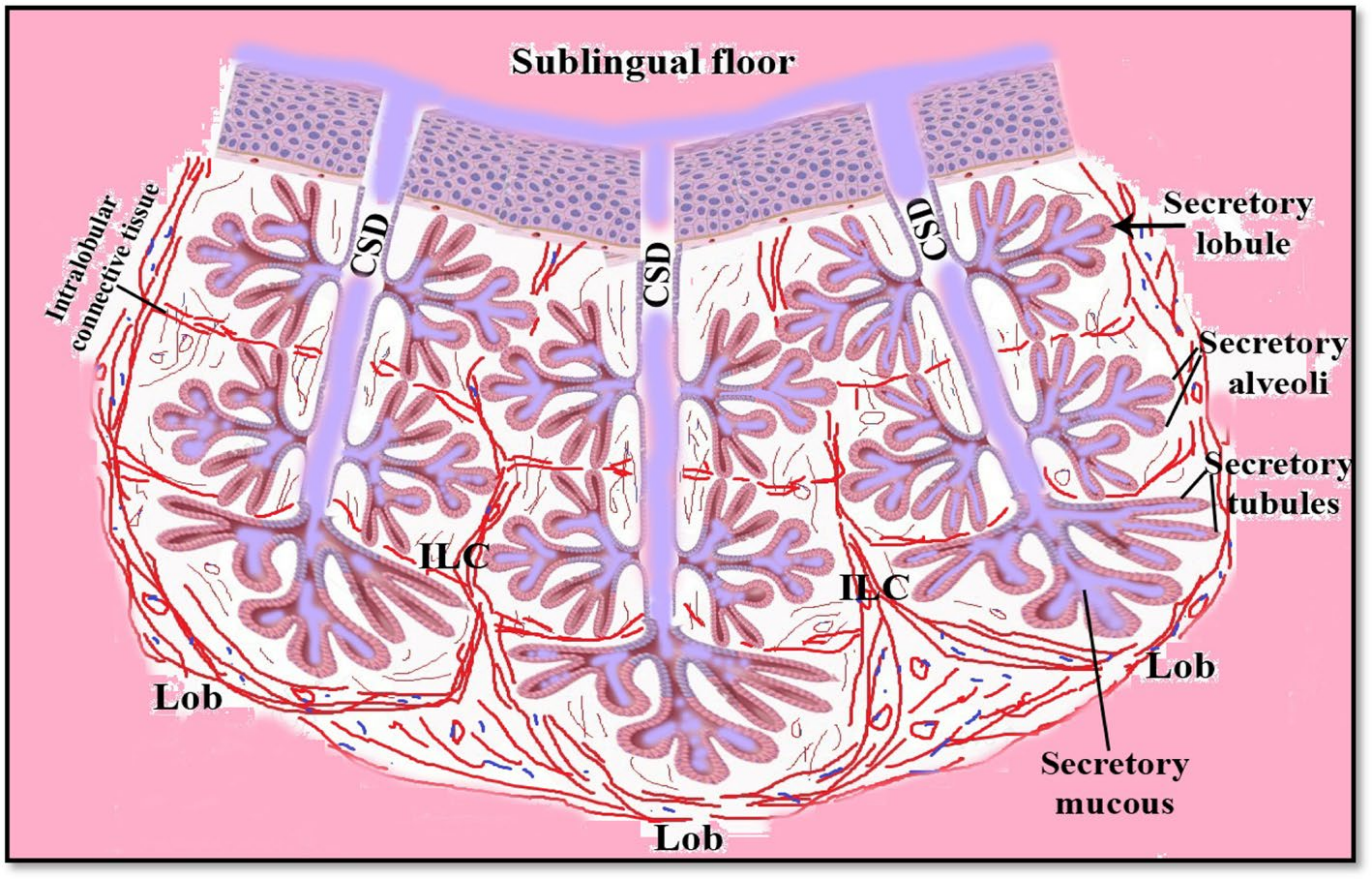
Schematic diagram showing the secretory units of the compound tubuloalveolar sublingual salivary glands. Note the common secretory duct (CSD), the interlobar connective tissue (ILC), and the glandular lobe (Lob)

The histochemical study (Table 2B) suggested that the mandibular salivary glands at 30-day-old quails gave a very strong positive reaction to all of the alcian blue, PAS, and combined AB/PAS stains (Fig. 13C-F). By the 60-day-old quail, these glands showed a strong positive reaction to AB stain and combined AB/PAS stains (greenish blue coloration only), but the glands gave negative results to PAS stain. Figure 14 is a schematic diagram showing the secretory units of the compound tubuloalveolar sublingual salivary glands. From previous findings, the oropharyngeal salivary glands, which were represented in the sublingual and mandibular salivary glands and their secretions, in addition to the taste bud system, had a great developmental change during the pre- and post-hatching ages that configured the feeding patterns of each bird.

## Discussion

Salivary glands and taste buds are unique specialized structures that play a vital role in mammals and birds [13, 14, 20]. However, little concern is shown by the researchers that help us to understand their role in the birds. These glands are called the sublingual and mandibular salivary glands, as anatomical nomenclature follows [21] in chickens. However [21–23], suggest that the Glandula sublingualis is synonymized to be Glandula submandibularis rostralis or mandibularis rostralis. While the Glandula mandibularis is synonymized to be (Gl. Mandibularis caudalis) or (Gl. Submandibularis caudalis). Moreover [3], divide them into lateral and medial mandibular glands in the emu. At this end [21, 22], discuss both glands deeply in chickens.

According to prehatching data, on days 6 and 10, respectively, the sublingual and mandibular salivary glands initially emerge as a thick invagination (a bud stage) of the sublingual floor and paralingual epithelium, in keeping with [4, 24]. While the current findings are compatible with [23]’s assumption that the primordia originate from the mouth floor epithelium that is expanded without branching at the 8-day quail stage. On days 10 and 11, of the incubation period, respectively, the sublingual gland’s canalization process begins and becomes clearly visible. Thus, the process by which the glandular lining changes from stratified to monolayer epithelium (simple glandular epithelium). On the thirteenth day before the hatching, the sublingual gland primordia begin to branch [23]. Observe that a branching mechanism exists in the lingual salivary gland that resembles the mandibular salivary glands but is different from the sublingual salivary glands. As the same developmental steps, the murine antenatal morphogenesis of the salivary glands branching is a clear example in prebud, initial, canalicular, and terminal bud stages [25, 26].

The sublingual salivary glands undergo cellular proliferation, quiescence, apoptosis, and cytodifferentiation events during all of their prenatal developmental stages. These events are triggered by unique growth factors, cytokines, and signaling pathways that targeted transcription factors, causing them to be upregulated both temporally and spatially [26–31]. Studies demonstrate that the development of sublingual glands is reliant on the communication between the mesenchymal and epithelial sides [32].

Similarly, the 13-day-old embryo’s glandular lumina contained cellular fragments and a secretory-like substance. AB or PAS stains give negative results, which is consistent with [33]. Furthermore [34], find that the stimulation of swallowed albumin at the 13-day-old chick embryo results in the release of an acidic secretion from the developing proventriculus, marking the start of the alimentary tract secretions at this critical age. Notable, the preglottal salivary glands in quails revealed a similar result [4].

Within the sublingual salivary glands, it indicates that the acini open directly into a large common wide secretory lumen of the glandular lobule, which served as a common excretory duct to open into the oral cavity. Notably, the secretory system is lined with the same cell type as the secretory end pieces: simple columnar cells that are transformed to low columnar cells to finally become stratified epithelium in agreement with [33, 34]. The secretory end pieces of the other salivary glands, like the mandibular salivary glands, drain their secretion within intralobuar ducts and then into the interlobar duct to the common secretory canal. Similarly [35], find that the cellular lining of the glandular duct and the central lumen is simple columnar with microvilli at the apical cellular portion in the posterior lingual salivary gland in quail. Also [3], support the presence of a central canal, but the glands are simple branched tubular in the emu, and the lining is simple ciliated columnar cells or pseudostratified columnar epithelium. While in the present study it is compound tubuloalveolar gland in agreement with [36] in rock dove. Furthermore, the sublingual salivary glands of mice consist of large and small ducts that ended in a single lumen-containing secretion [25, 26, 29].

It is clear from gross and histological data that the sublingual gland lobation is different from [21] in chickens, who assume that the gland consisted of 5–7 lobes. Our findings reveal that the lobation number is varied bilaterally, and unilaterally in relation to the location.

The current study shows that the mandibular glands are paired compound branched tubulo-alveolar glands within the submucosa of the paralingual floor of the mouth. Whereas, it extends from the caudal of the frenulum linguae to the tips of the gland sublingualis. Their orifices open into the bottom of the paralingual groove. Accordingly, these results agree with [3, 9, 21, 35, 37]. However [38, 39], assume the mandibular salivary glands to be a part of the gland sublingualis. The glands are supported with thick connective tissue stroma that is compatible with [3].

Interestingly, the taste buds are two types; the larger one is associated with the salivary glands opening, and the smaller one, called surface taste buds, lies free on the oral surface mucosa, corresponding to the findings in chicken [37, 40, 41], and in emu [3]. While the preglottal salivary gland opening widths are 25.5 μm and 80.4 μm at 14- and 30-day-old quail chicks, respectively [4]. However [42], find that taste buds parameters in chicken are (40–70 μm) in width and (70–120 μm) in height. While the taste pore is (3–7 μm) in width. Although the openings of the salivary glands are greater than (10 μm) in diameter and are observed near the taste buds, other authors’ data reveal that taste pores width is equal to (5–10 μm) [40] or (6 μm) [37].

The present study (Table 1) shows the average diameter in (um) of the sublingual salivary gland openings (SGO), the taste bud openings (TBO), and the taste buds surface area (TBA) which is represented by (um^2^) in quails. The taste buds were categorized into three size groups: larger, moderate, and smaller, and according to the position of taste buds associated with salivary gland opening and surface epithelial one. Also, taste pores varied from (8.2–12 μm). Salivary glands act as a flusher by mucous secretion for taste buds associated with their opening, like Ebner’s glands in mammals. Our studies found two types of taste buds that revealed two different food discrimination and ecological patterns of the avian species. Therefore, there are two types of receptors for sugar-rich sources (e.g., nectar or sweet fruits) as the principal nutrient sources in hummingbirds, sugarbirds, and sunbirds, related to type 1 receptor taste buds, while two taste receptors (T2R) play a primary defense function to prevent the ingestion of potential toxic compounds, presumably by eliciting bitterness or a similar unpleasant sensation [14].

From our histological results, we find the sublingual and mandibular salivary glands secrete mucous in agreement with [43]. The glands are strongly positive to PAS stain, but they are weakly positive to AB stain on the hatching day of chicks. The glands react negatively to PAS but strongly to AB stain at 30- and 60-days post-hatching [44, 45]. find that the posterior lingual salivary glands are stronger positive for neutral mucopolysaccharides than the anterior one in both Common Myna and red jungle fowl. However, there is no mucosubstance in the lingual salivary glands of the little erget [2, 35, 46]. support the presence of sialo-glycoconjugates within the preglottal and the lingual salivary glands in Japanese quails. Concurrently, the same result is approved in the salivary glands of chickens [47, 48].

In addition [12, 35], support the presence of sialo-glycoconjugates within the preglottal and the lingual salivary glands in Japanese quails. Also, this is stated in the salivary glands of chickens by [47, 48]. However, the palatine salivary glands of chickens produce glycoproteins, sulphomucins, and carboxymucins, which are rich in sialic acid and show more PAS and alciniophilic-containing metachromatic granules [49]. Of note [50], suggest that the lingual glands are more acidic with sulphated or acidic sialomucin. The double differential staining of alcian blue (AB) and PAS stains differentiates between acidic glycoconjugates stained (blue) with AB and neutral ones stained (magenta) with PAS in agreement with [45] in chicken. We conclude that the secretory mucous takes the fate from neutral to acidic result.

A tentative interpretation of type of the lingual gland secretions in little egret can be made according to the classification of mucosubstances [51–53]. The lingual salivary gland of little egret is exhibited to be alcianophilia and reacts positively to the different techniques that are employed for protein detection. These glands were confirmed to be of mucoserous type. The lingual glands of chicken [54], penguin [55], white-cheeked bulbul [56], and Eurasian collared dove [57] are reported to be of mucous type; however, the quail [12] and the little egret [46] have a mixture of serous and mucous type salivary glands. The secretory products of the lingual salivary glands of these species of birds, as seen in the present study, form a blend of mucoserous secretions that contain sialomucins, sulfomucins, and proteins [46]. To illustrate that our result finds the glands are well developed in quails and take acidic reactions with age advancing as [55] findings. As our findings, show the secretory units of the compound tubuloalveolar sublingual salivary glands (Fig. 14).

## Conclusion

In summary, the sublingual salivary glands and mandibular salivary glands show different histochemical changes in mucous, where both glands were the basic feeding adapting units among the mucous salivary gland system. Whereas, the glandular system gives flexibility in various food intakes and is responsible for the softening of food chews. Hence, many immunostaining, genetic assessment, scanning, and transmission electron microscopy investigations are needed for further future explanation of the salivary gland and taste bud receptor types and roles in the avian feeding habits and ecological patterns.

## Supplementary Information

Supplementary Material 1.

## Data Availability

The data that support the findings of this study are available on request from the corresponding author.
