# Supplementary material for: Developmental studies of the sublingual and mandibular salivary glands in Japanese quails (Coturinx coturinx japonica)
Source: BMC Vet Res. 2024 Nov 12;20:512. doi: 10.1186/s12917-024-04355-7 (PMC11555808; doi:10.1186/s12917-024-04355-7)
Supplement: Supplementary file 1 — Supplementary Material 1. [file 12917_2024_4355_MOESM1_ESM.docx]

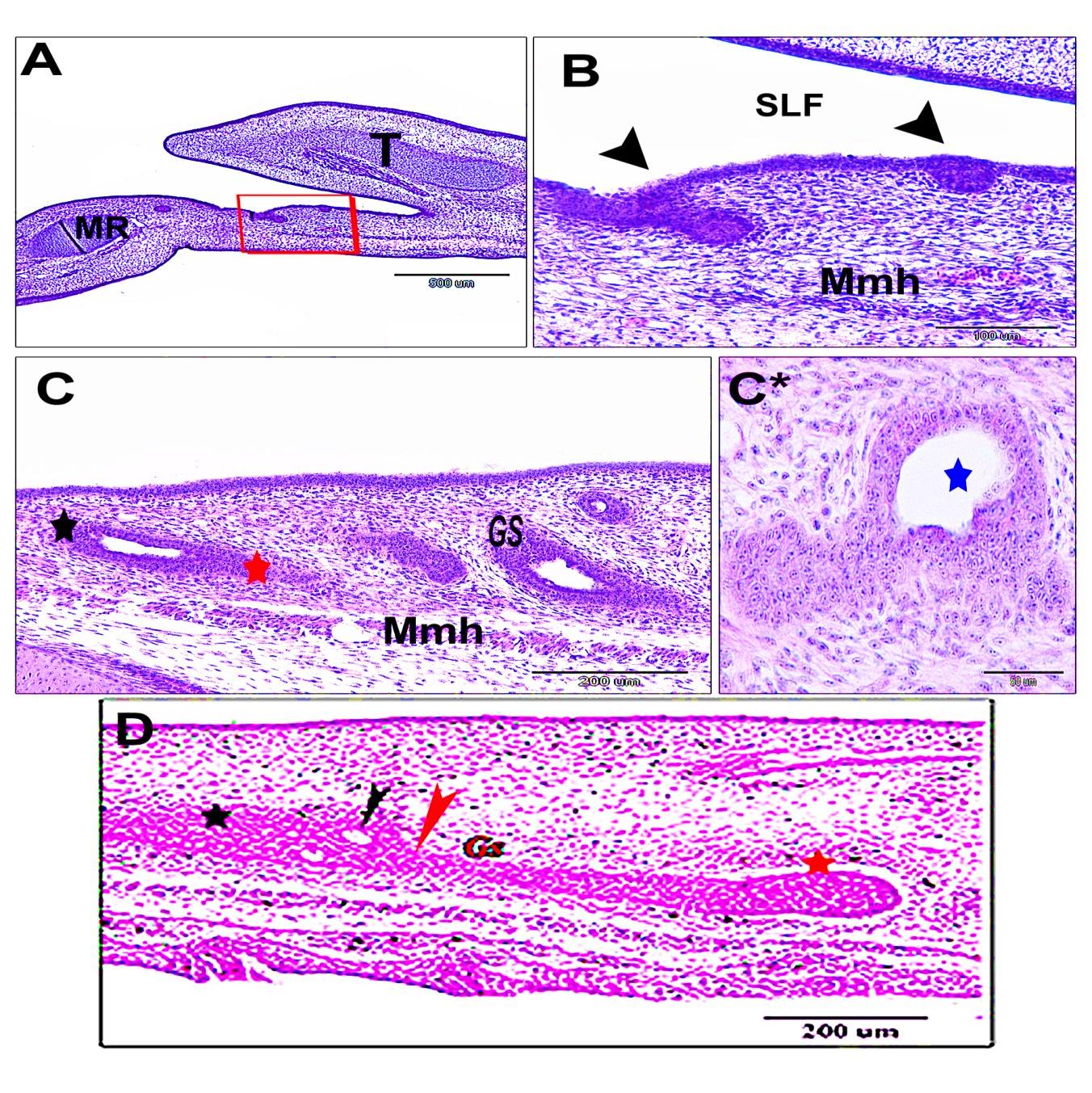


Fig. (1): Photomicrographs of the sublingual floor: (A & B): Sagittal sections of a 6-day old quail embryo, showing the primordia of the sublingual salivary glands (Rectangular shape, Fig. 1A). Note: the epithelial thickening of the sublingual floor mucosa (epithelial placode) (black arrowheads, Fig. 1B). (SLF) sublingual floor, (T) tongue, (MR) mandibular ramus, muscle mylohyoideus (Mmh) (C): Sagittal sections of 10-day old quail embryo showing the glands sublingualis (Gs) have a canalized cord like shape with two ends; rostromedial wide part (black star), and caudolateral narrow part (red star). Notice: the muscle mylohyoideus (Mmh). (C*): Cross section showing canalization (blue star) within the gland and stratified lining epithelium of the same age. (D): 11-day old quail embryo showing the extension of the cord of the gland sublingualis (Gs) with its rostromedial wide part (black star) covered by concentric layer of mesenchyme and caudolateral narrow part (red star) uncovered by mesenchyme, and central constricted part (red arrowhead) small spaces have been shown (black arrowhead). H & E, Scale bars; A: 500 µm, B: 100 µm, C: 200 µm, C^*^: 50 µm, D: 200 µm.


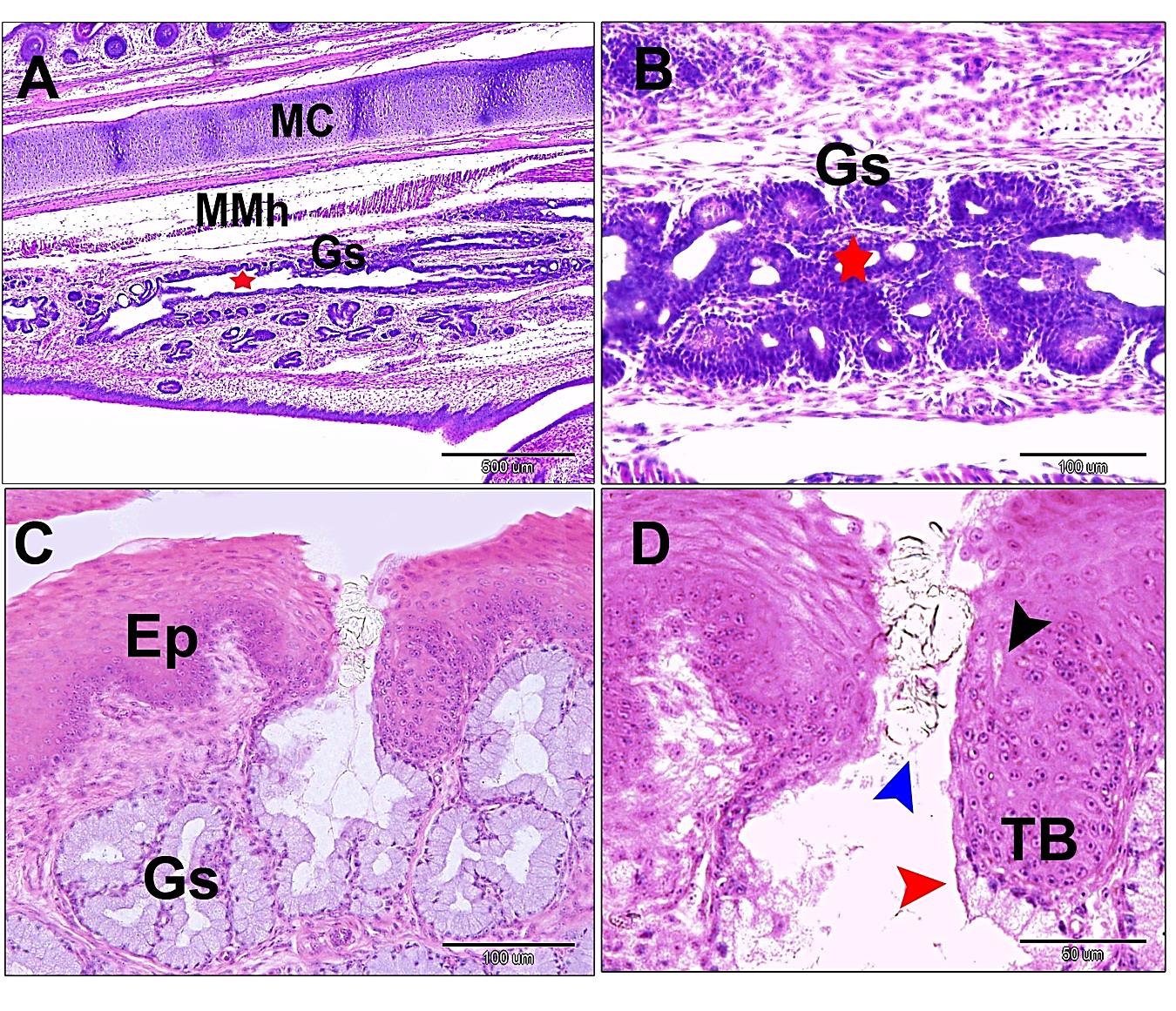


Fig. (2): Photomicrographs of frontal sections in the sublingual floor of 13-day old quail embryo (A): Showing straight common canal (red star) along the gland sublingualis (Gs). Note: the muscle myelohyoid (Mmh) and Meckelian cartilage (MC). B: Showing the presence of cellular exfoliation (red star) of the gland sublingualis. (C & D): Cross sections of a hatching quail chick; C: Showing the well-organized gland sublingualis (Gs) opening in the surface epithelium (Ep; stratified squamous epithelium non cornified). D: Showing the simple columnar glandular epithelium (red arrowhead), more infoldings in secretory end pieces and the secretory materials (Blue arrowhead) of the gland sublingualis. Note the taste bud (TB) with taste pore (black arrowhead). H & E, Scale bars; A: 500 µm, B: 100 µm, C: 100 µm, D: 50 µm.


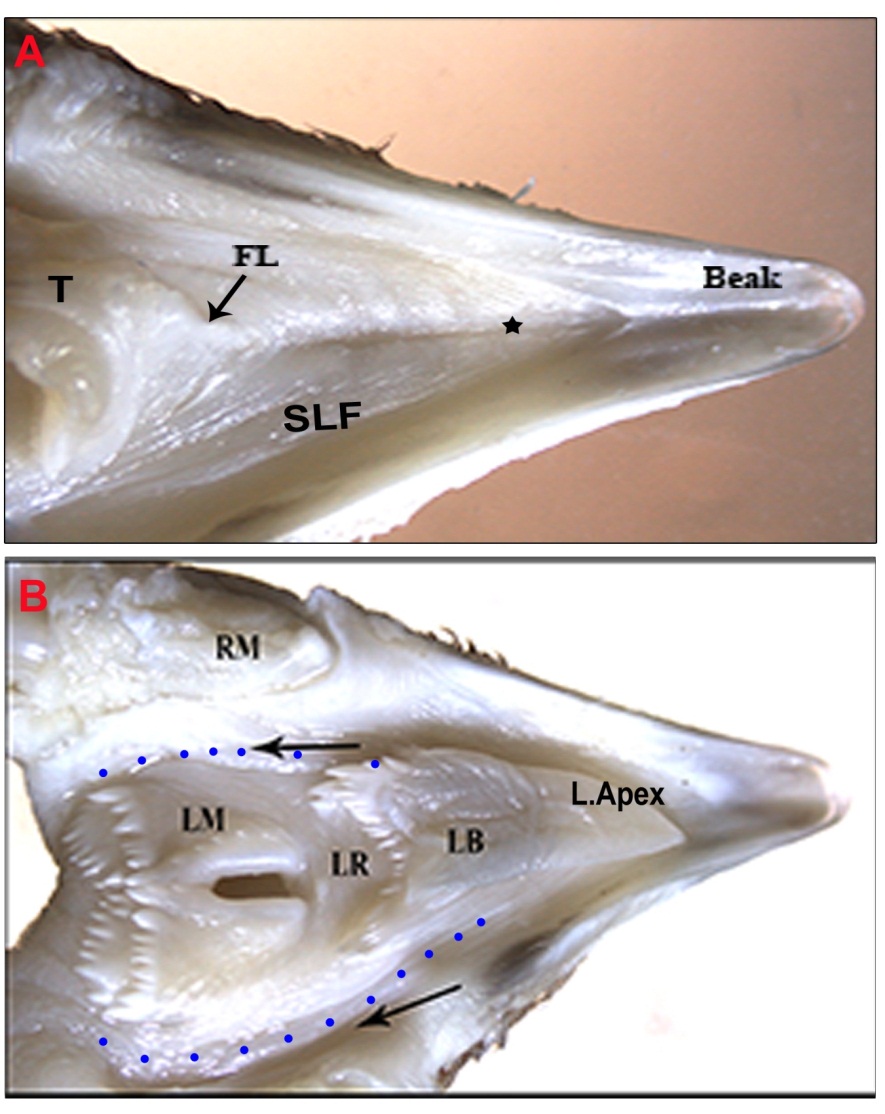


Fig. (3): Photographs of a dorsal view of the oropharyngeal floor of a 14-day old quail chick: (A) Showing sublingual floor (SLF) after tongue (T) reflection, and prefrenular median sulcus where sublingual salivary glands open (black star). Note, frenulum linguae (FL). (X 6.3). (B) Photograph showing pharyngeal floor parts of paralingual grooves (black arrows) and distribution of mandibular salivary glands (blue dots). Note, Ramus mandibularis (RM), lingual apex (L.Apex), lingual body (LB), Lingual root (LR), and laryngeal mound (LM). (X 6.3).


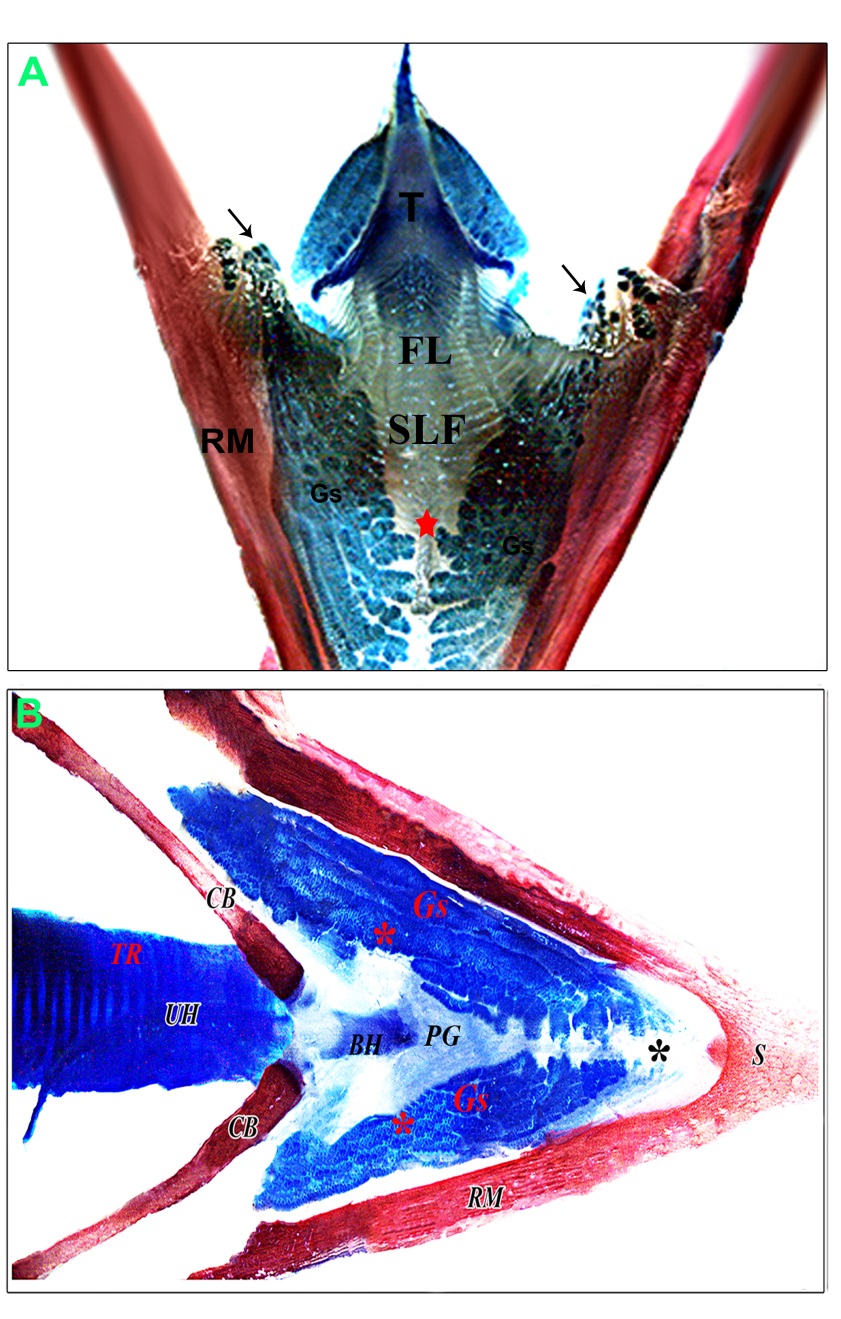


Fig. (4): Photographs of the gross staining (after maceration) of 14-day old quail chick (A) dorsal view of the oral floor after tongue (T) reflection showing sublingual floor (SLF), and prefrenular median sulcus where sublingual salivary gland open (red star). Note, frenulum linguae (FL). (Alzarin red & alcian blue stains, X 6.3). (B) ventral view of the lower beak of a 14-day old quail chick showing gland sublingualis (Gs) extends from symphysis mandibularis (S) beyond the first parts of the ceratobranchialia (CB), ramus mandibularis (RM). Note the basihyoid (BH), Paraglossal (PG), trachea (TR), urohyale (UH), and central constricted part (red asterisks). Sublingual groove (black asterisk). (Alizarin red & alcian blue X 6.3).


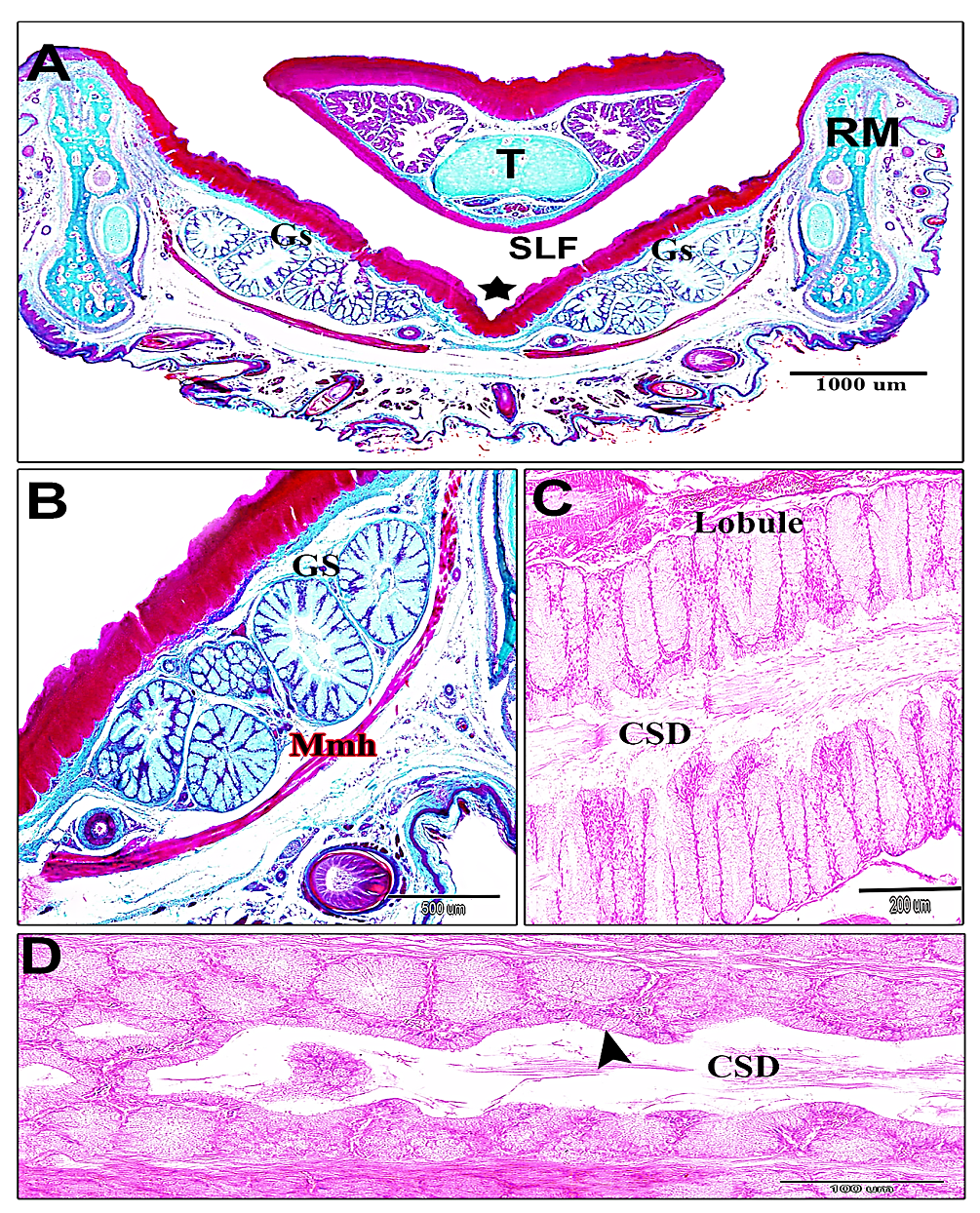


Fig. (5): Photomicrographs of oral floor of a 30-day old quail: (A & B): Cross sections showing the paired gland sublingualis (Gs) which composed of variable 5-7 lobules in each side at the oral part; lying in submucosa and supported ventro-laterally by muscle mylohyoideus (Mmh). Note: median sulcus (black star), ramus mandibularis (RM), sublingual floor (SLF) and tongue (T). (Crossmon’s trichrome stain, Scale bars; A: 1000 µm, B: 500 µm). (C & D): Frontal sections showing the lobular structure of gland sublingualis composed of compound tubuloalveolar secretory units open into common secretory duct (CSD) which lined by simple columnar epithelium (black arrowhead). (H & E stain, scale bars; C: 200 µm, D: 100 µm).


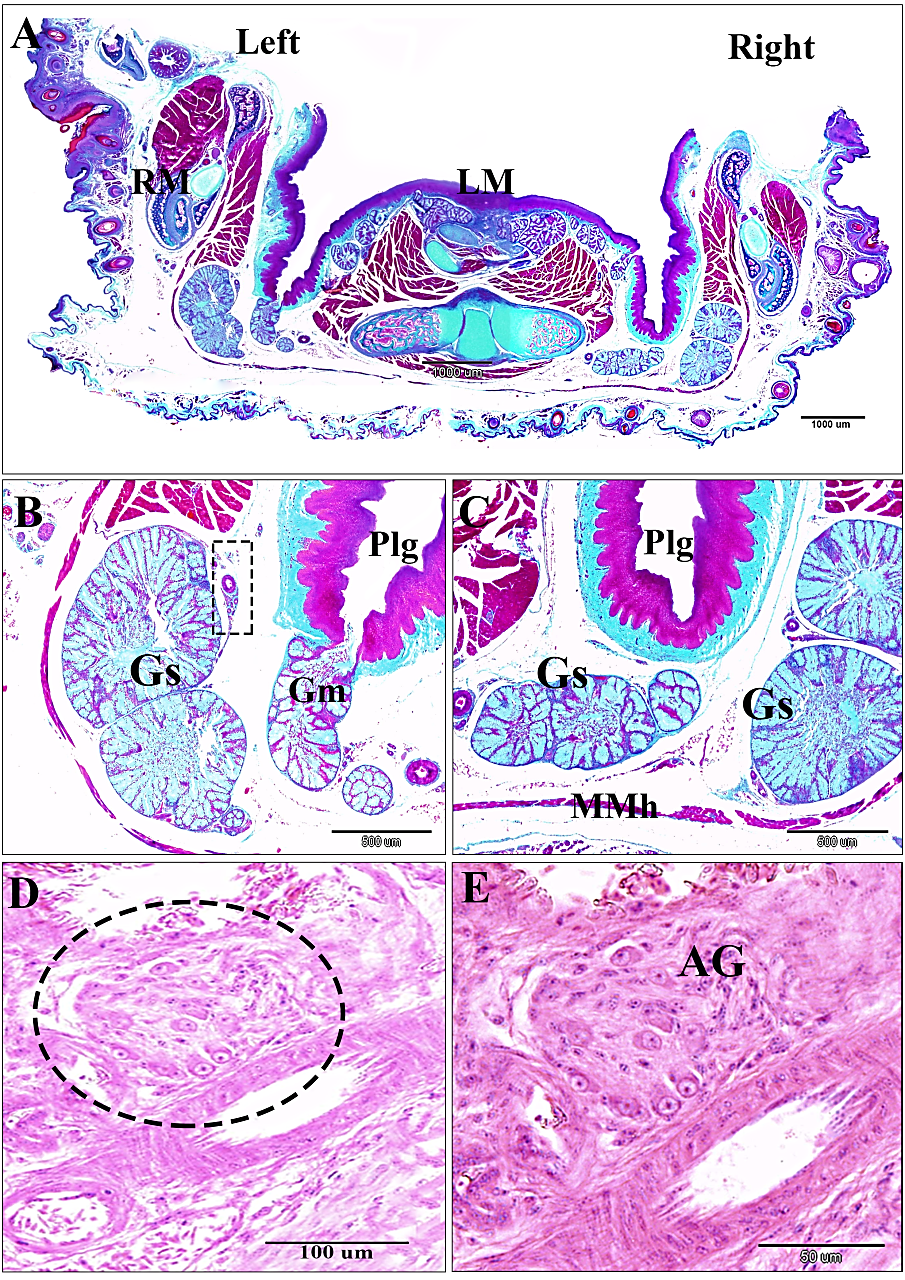


Fig. (6): Photomicrographs of pharyngeal floor of a 30-day old quail. The left side showing gland sublingualis (Gs) consist of 3 lobes (A & B) while the right side showing 5 different sized lobes; 2 larger ones, 2 moderate ones and a smaller one in (A & C). Note: muscle mylohyoideus (Mmh), paralingual groove (Plg), gland mandibularis (Gm) and ramus mandibularis (RM), laryngeal mound (LM). (Crossmon’s trichrome stain, scale bars; A: 1000 µm, B & C: 500 µm). (D & E): Autonomic ganglion (AG) adjust to the glands sublingualis. (H & E stain, scale bars; D: 100 µm; E: 50 µm).


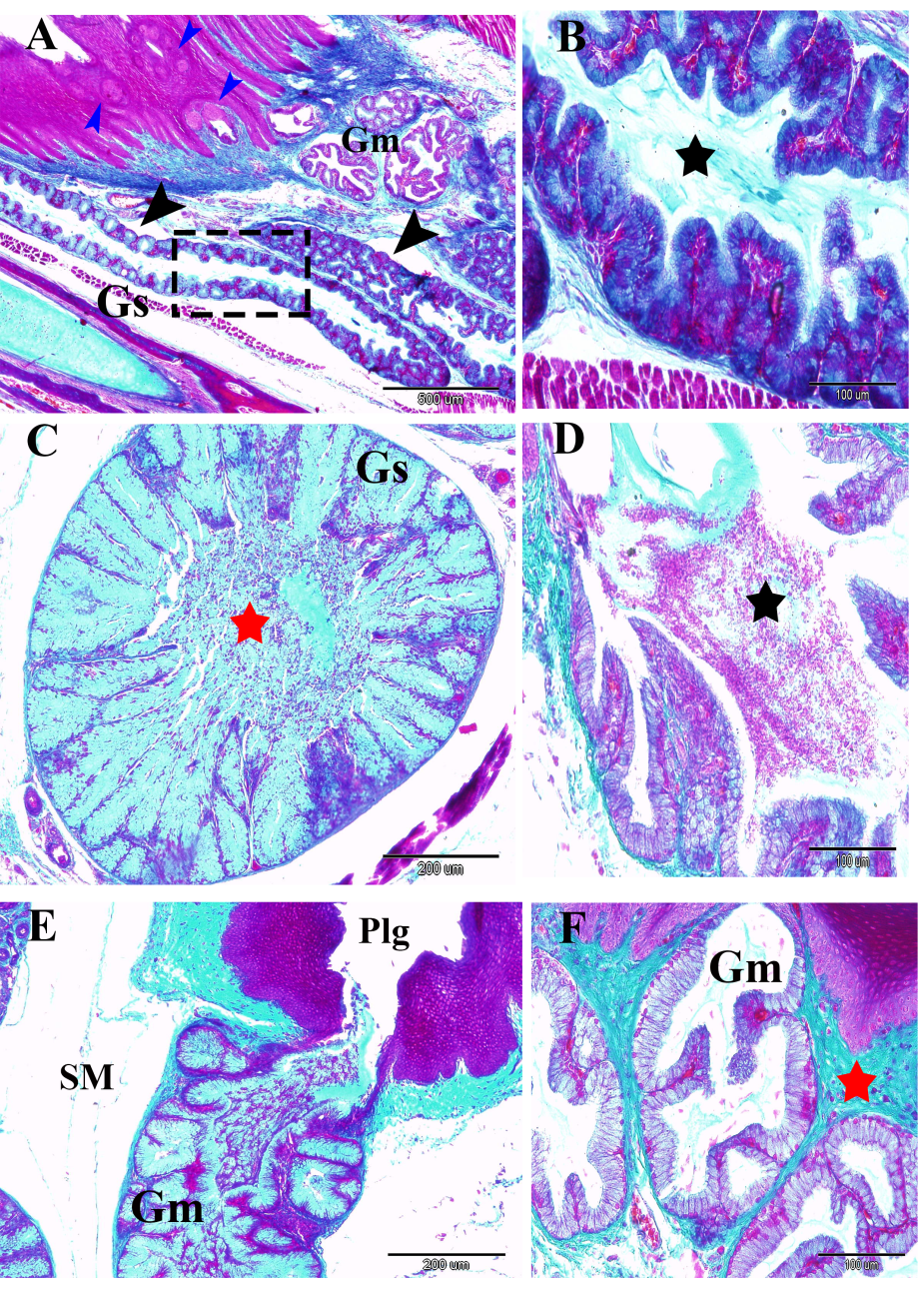


Fig.(7): Photomicrographs of different sections in the oropharyngeal floor of a 30-day old quail chick. (A): Frontal section showing many taste buds within the epithelium (blue arrowheads), the gland sublingualis (black arrowheads), the lobar structures of the gland sublingualis (black dash rectangular shape). (B): The lobar structure of the gland sublingualis with a central lumen (Black star) (C): Cross sections of the gland sublingualis (Gs) at the pharyngeal level with wide central lumen (red star) filled with secretion. (D): Apocrine secretion, the cellular content depris (red color) and secretion (greenish color). (E): Apocrine secretion of the gland mandibularis (Gm) poured into paralingual groove (Plg) with cellular contents and secretions. Note the submucosa (SM). (F): Secretory endpieces with thick connective tissue septa (red star) of the gland mandibularis (Gm). (Crossmon’s trichrome stains, scale bars; A: 500 µm, B, D & F: 100 &µm C & E: 200 µm).


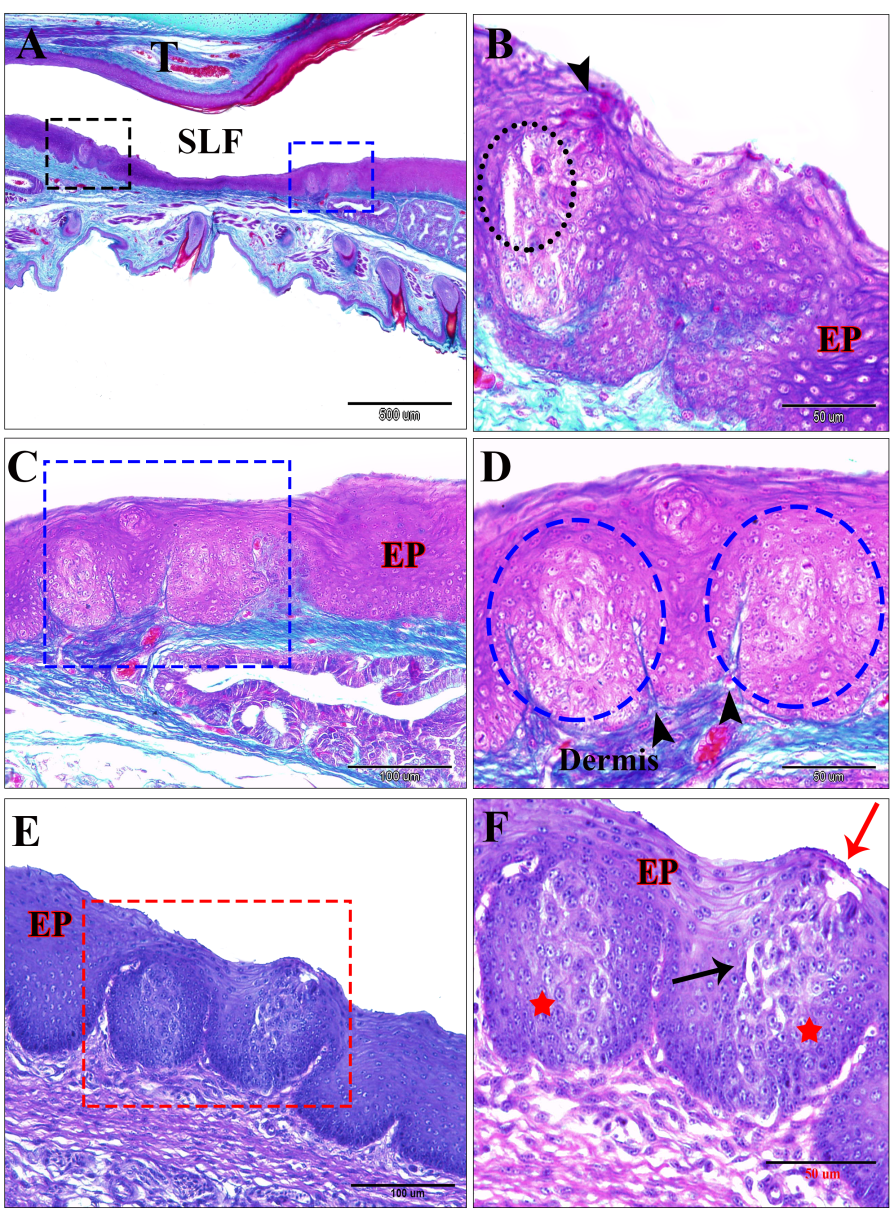


Fig. (8): Photomicrographs of different sagittal sections in the quail oropharyngeal floor. (A-D): Photomicrograph of a sagittal section oropharyngeal floor of 14-days old quail chick showing the types of the barrel shaped taste buds with taste pores. (A): Taste buds associated salivary glands opening (black rectangular shaped) and surface epithelial taste buds (blue rectangular shaped). Note the tongue (T) and sublingual floor (SLF). (B): Showing taste pores (black dotted circle), dark stained cells (black arrowhead) and epithelium (Ep). (C & D): Surface epithelial taste buds (blue dotted circle), with deep dermal papillae (black headarrows). (Crossmon’s trichrome stains, scale bars; A: 500 µm, B, D: 50 µm & C: 100 µm). (E & F): Photomicrograph of a sagittal section oropharyngeal floor of a 60-days old quail showing the opening of the gland sublingualis (red arrow) arose from the epithelium of the sublingual floor, taste bud (red rectangle), taste pores (black arrow), the basal epithelial highly mitotic cells (red star) and the surface epithelium (Ep). (PAS & Hx stain, scale bars, E: 100 µm & F: 50 µm).


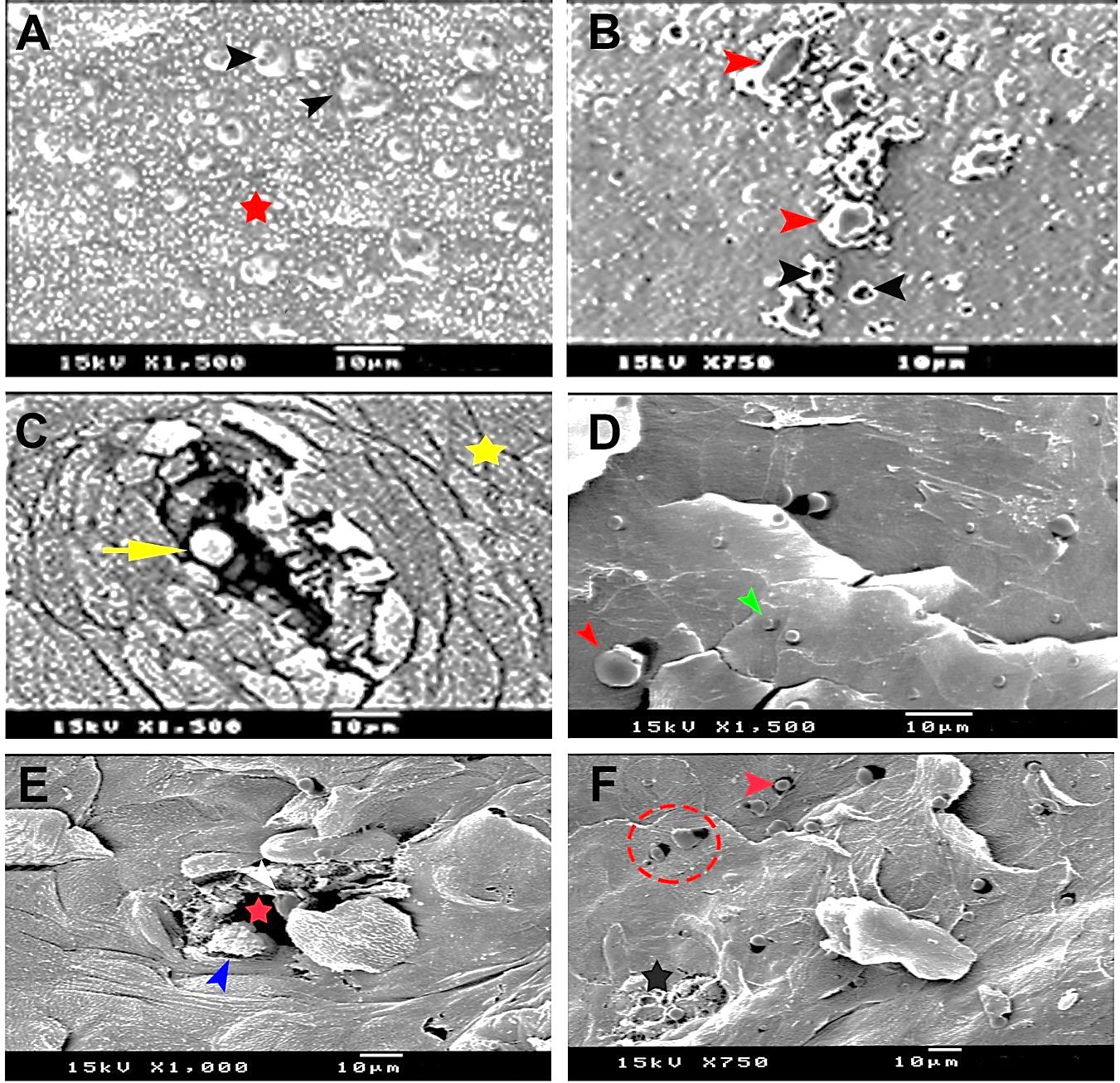


Fig. (9): Scanning electron micrographs of sublingual floor surface (A): 8 day of incubation, showing dome-shaped mushroom-like taste buds (black arrowheads) along the sublingual floor (red star) (X 1500). (B): 10 day of incubation, showing the opening of the sublingual salivary glands (black arrowheads) with mushroom-like taste buds associated with salivary glands (red arrowheads). (X 750). (C): 13 day of incubation showing the opening of the sublingual salivary glands with no secretion could be observed. Note taste bud associated salivary gland (yellow short arrow), surface epithelium with elongated flat cells (yellow star) (X 1500). (D): 30 days old chick showing the different sized taste buds along the sublingual floor epithelium arose among the epithelial scales; larger ones stuck with a little mucous and guarded the salivary gland opening (red arrowhead) and smaller ones, were the surface taste buds (green arrowhead). (E & F): 60 days post-hatching quail; (E): Showing the sublingual salivary gland opening (red star), mucous debris adjacent to the opening's rim (blue arrowhead), large-sized taste buds associated with salivary gland (white arrowhead) (X 1000). (F): Showing the taste buds associated with the sublingual surface surrounded with pits (red dotted circle and red arrowhead), others associated with mucous (black stars). Note the decrease in the number of the small surface taste buds type.

V


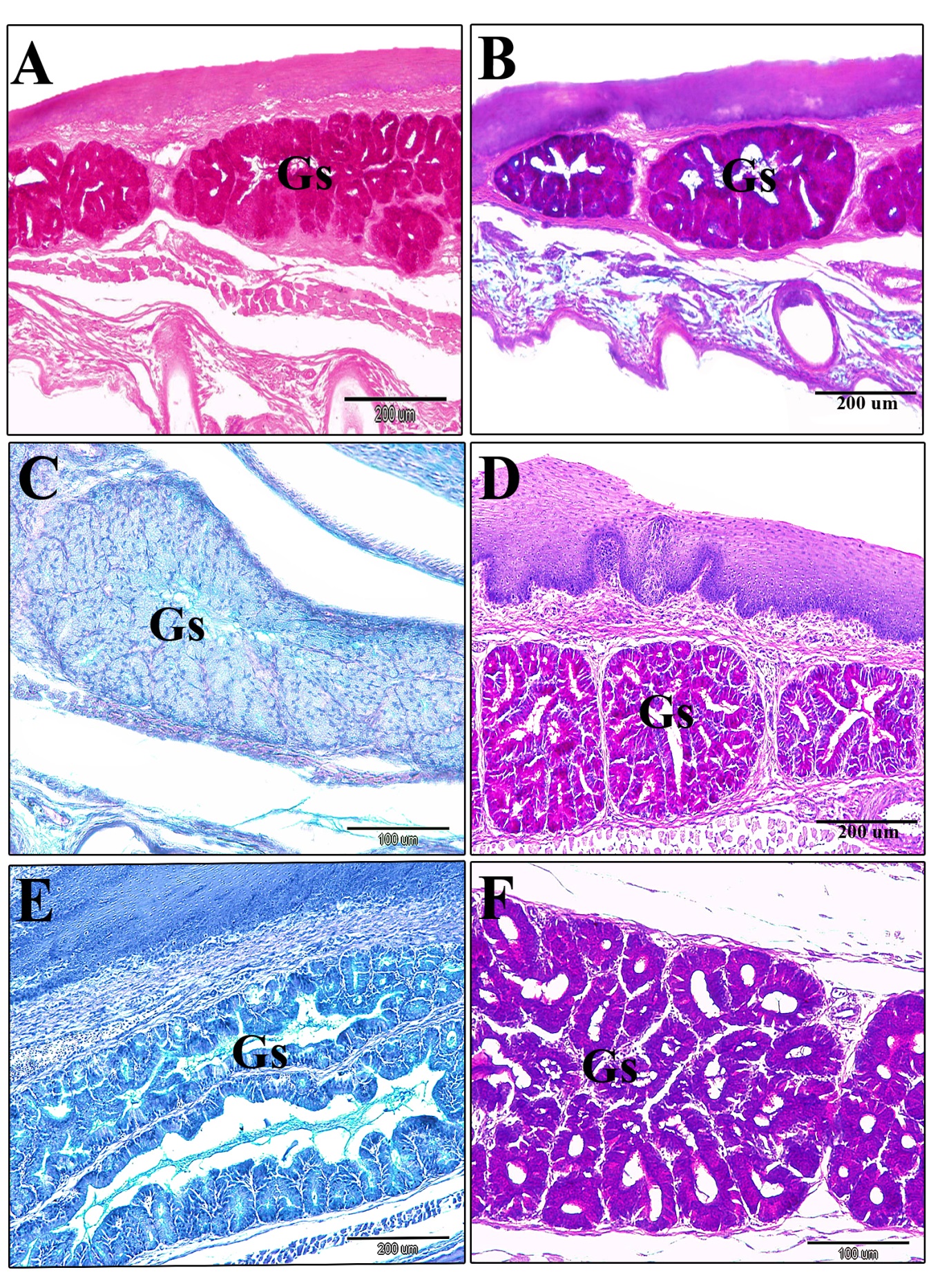


Fig. (10): Photomicrographs of sagittal sections in the oropharyngeal floor of a newly hatching quail chick. (A): Showing the gland sublingualis strong positive to PAS stain. (PAS stain, scale bar 200 µm). (B): Showing the gland sublingualis strong positive to combined AB/PAS stains. (AB/PAS stains, scale bar 200 µm). (C): Showing the gland sublingualis weakly positive to AB stain, scale bar 100 µm). (D): 7-day old quail chick showing the gland sublingualis strong positive to PAS stain. (PAS stain, scale bar 200 µm). (E): A frontal section of a 7-day old quail chick showing the gland sublingualis moderate positive to AB stain. (AB stain, scale bar 200 µm). (F): A sagittal section of a 7-day old quail chick showing the gland sublingualis strong positive to combined AB/PAS stains (purple coloration). (AB/PAS stains, scale bar 100 µm).


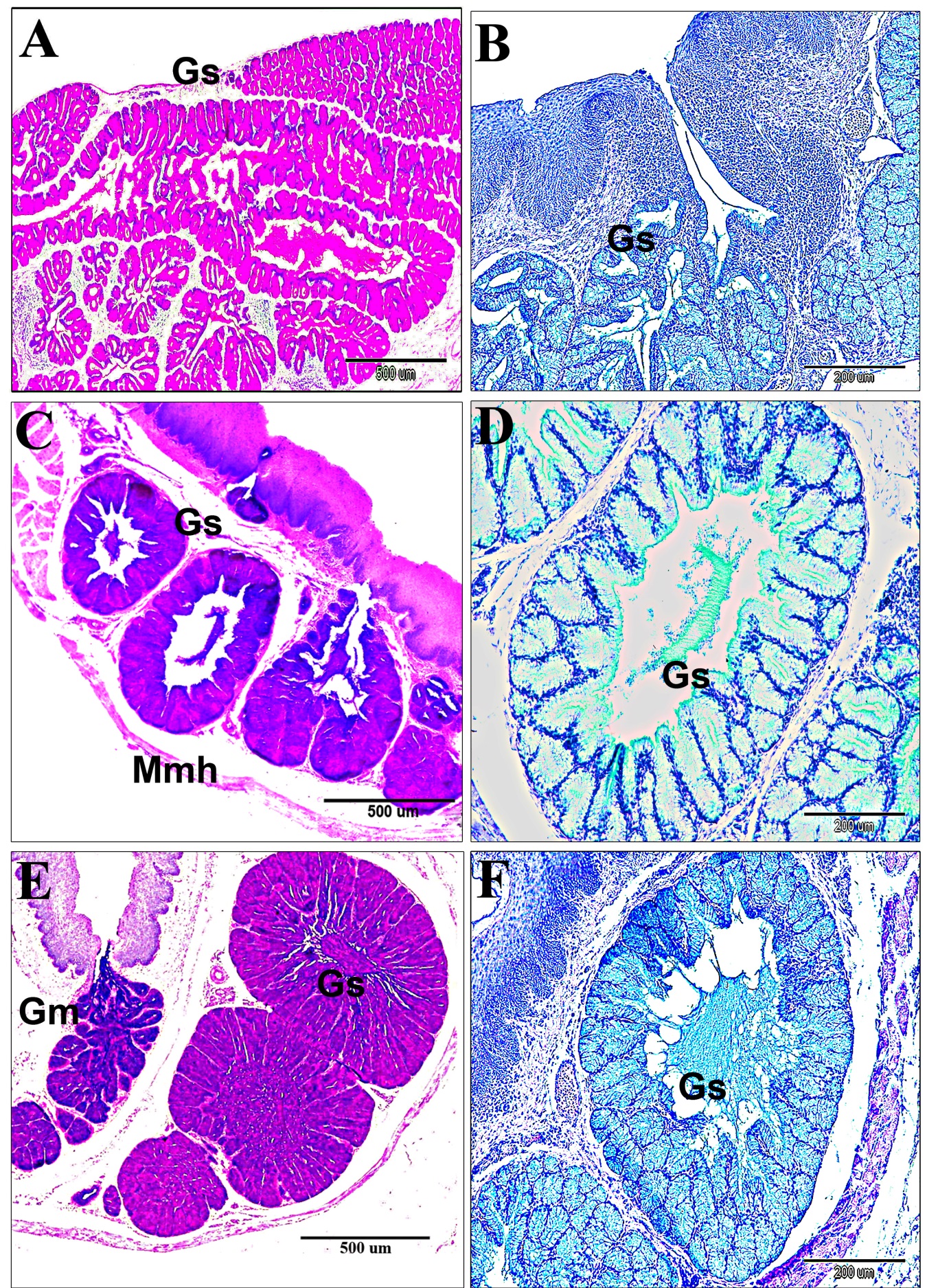


Fig. (11): Photomicrographs of the oropharyngeal floor of a 14 day (A & B & C); a 30-day (D & E) and a 60-day (F) old quail chick. (A): A frontal section showing the gland sublingualis very strong positive to PAS stain. (PAS stain, scale bars 500 µm). (B): Showing the gland sublingualis moderate positive to AB stain (AB stain, scale bars 200 µm). (C): Showing very strong positive to combined AB/PAS stains. (AB/PAS stain, scale bars 500 µm). (D & E): Photomicrograph of a cross-section in the oropharyngeal floor showing the gland sublingualis strong positive to AB stain & very strong positive to combined AB/PAS stains (purple coloration) respectively. (D: AB, scale bars 100 µm E: AB/PAS, scale bars 500 µm). (F): Cross section showing the gland sublingualis very strong alcinophilic to AB stain and the same result with combined AB/PAS stains. (AB stain, scale bar 200 µm).


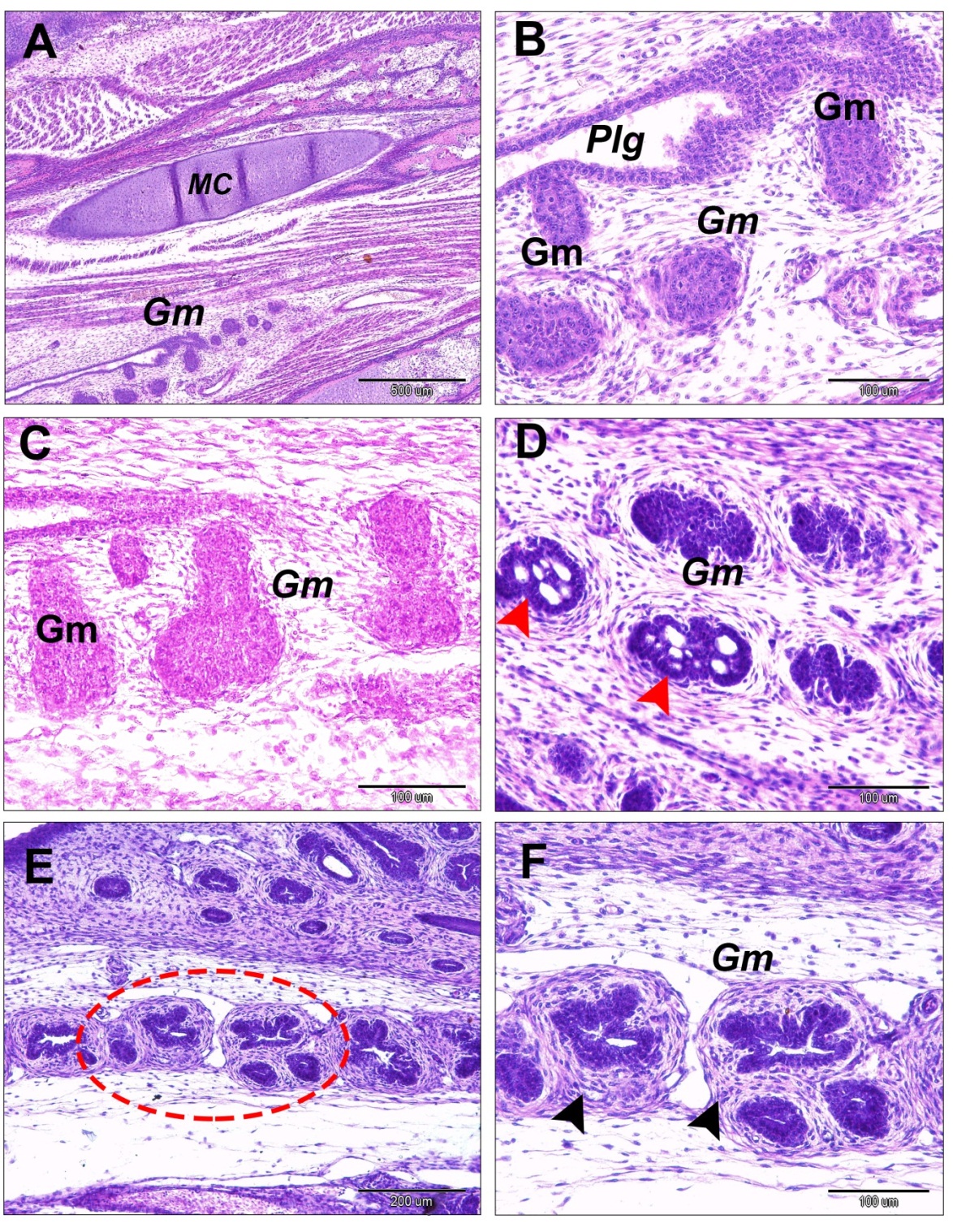


Fig.(12): Photomicrographs of frontal sections in the oro-pharyngeal floor: (A & B): Showing the gland mandibularis (Gm) buds and cords of 10-days old quail embryo originated from epithelial of the paralingual groove (Plg). Note the Meckelian cartilage (MC). (C): Expansion of the cord of 11-days old quail embryo. (D): Canalization of the expanded cord of 12-days old embryo and thin mesenchymal interaction (red arrowheads). (E & F): Thick concentric mesenchymal layers surrounded the branched cord of 13-days old embryo with more branched endpieces (black arrowheads). (H & E stains, scale bars; A: 500 µm, B, C, D & F: 100 µm, E: 200 µm).


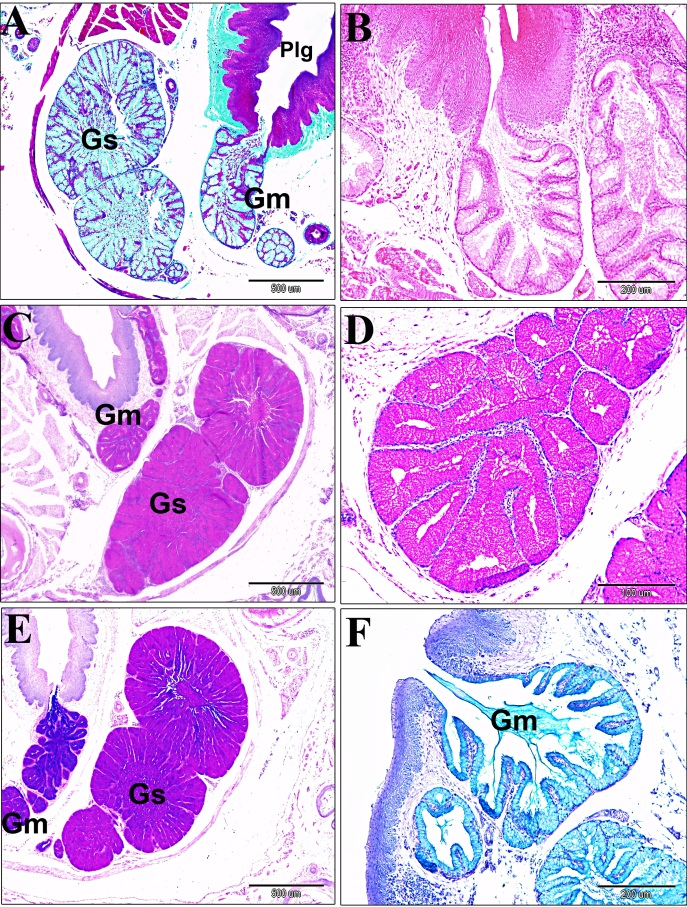


Fig. (13): Photomicrographs of transverses sections in the oro-pharyngeal floor of a 30-day old quail (A): Showing the gland mandibularis (Gm) lies dorsomedially to gland sublingualis (Gs) and opens ventrally into paralingual groove (Plg). (Crossmon’s trichrome, scale bar 500 µm). (B): Showing the gland mandibularis (Gm) composes of compound tubule-alveolar secretory endopices lies in the submucosa. (H & E, scale bar 200 µm). (C & D): Showing the gland mandibularis (Gm) and the gland sublingualis (Gs) very strong positive to PAS stain (PAS, scale bar; C: 500 µm, D: 100 µm). (E): Showing the gland mandibularis (Gm) and the gland sublingualis (Gs) very strong positive to combined AB/PAS stains. (AB/PAS stains, scale bar: 500 µm). (F): Showing the gland mandibularis (Gm) very strong positive to AB stain. (AB stain, scale bar: 200 µm).


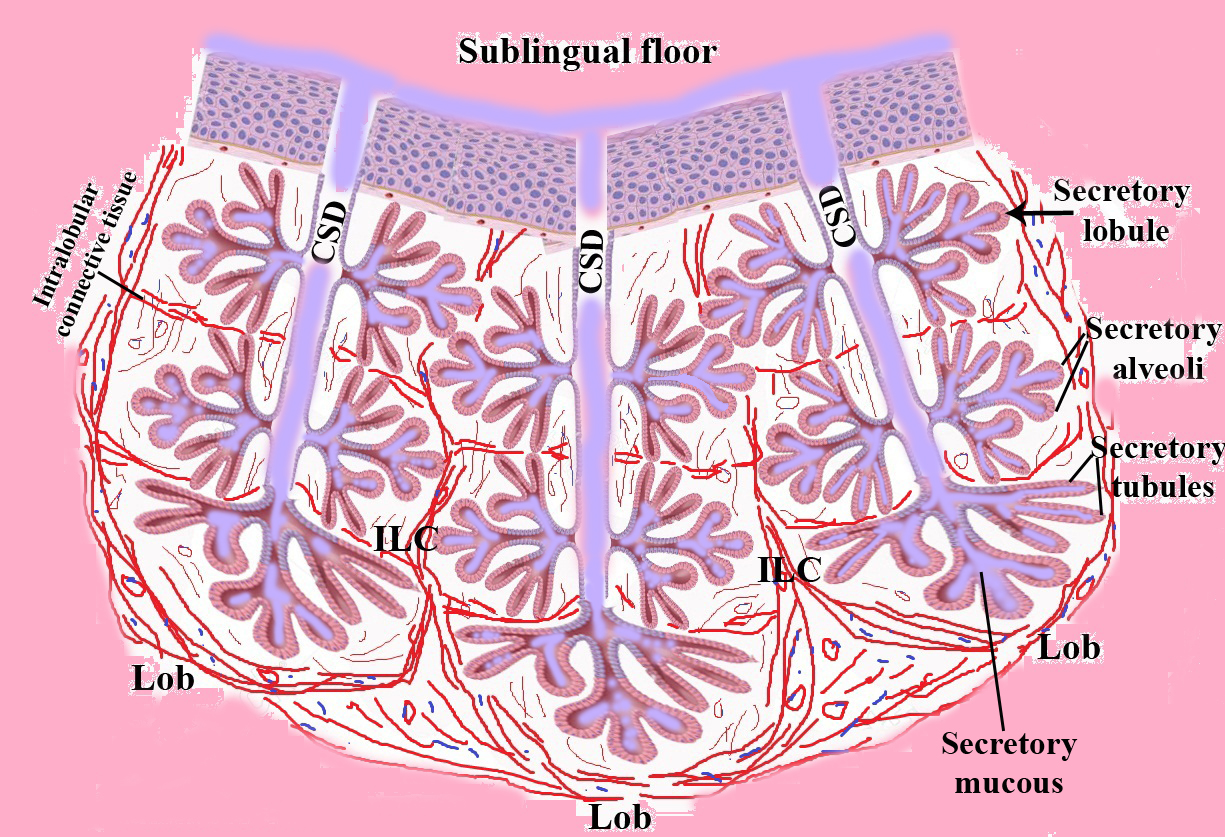


Fig. (14): Schematic diagram showing the secretory units of the compound tubuloalveolar sublingual salivary glands. Note, the common secretory duct (CSD), the interlobar connective tissue (ILC), and the glandular lobe (Lob).
